# Discontinuation risk from adverse events: immunotherapy alone vs. combined with chemotherapy: a systematic review and network meta-analysis

**DOI:** 10.1186/s12885-024-11897-4

**Published:** 2024-01-30

**Authors:** Sangwon Shin, Jimin Moon, Chiyoon Oum, Seulki Kim, Soo Ick Cho, Yoojoo Lim, Chan-Young Ock, Seunghwan Shin

**Affiliations:** Lunit, 4th to 8th floors, 374, Gangnam-daero, Gangnam-gu, Seoul, Republic of Korea

**Keywords:** Adverse drug event, Chemotherapy, Immunotherapy, Network meta-analysis, Treatment-related adverse event

## Abstract

**Background:**

While immunotherapy combined with chemotherapy (Chemo-IO) is generally recognized for providing superior outcomes compared to monotherapy (mono-IO), it is associated with a higher incidence of treatment-related adverse events (TRAEs), which may lead to treatment discontinuation. In this study, we compared the rates of treatment discontinuation between mono-IO and Chemo-IO as first-line treatments for various solid tumors.

**Methods:**

We systematically reviewed clinical trials from databases (PubMed, Embase, Cochrane Library, and an additional source) published from January 1, 2018, to July 10, 2023. We included phase III randomized controlled trials (RCTs) that utilized immunotherapy agents in at least one arm as first-line treatments for a variety of solid tumors. Data extraction followed the Preferred Reporting Items for Systematic Reviews (PRISMA) extension statement for network meta-analysis. A random effects model was used for the network meta-analysis, with the risk of bias assessed using the Cochrane risk-of-bias tool II. The primary outcomes encompassed treatment discontinuation rates due to TRAEs among patients who underwent immunotherapy, either alone or combined with chemotherapy, for various solid tumors. Pooled relative risks (RRs) with 95% confidence intervals (CIs) were calculated to compare between treatment groups.

**Results:**

From 29 RCTs, a total of 21,677 patients and 5 types of treatment were analyzed. Compared to mono-IO, Chemo-IO showed a significantly higher rate of discontinuation due to TRAEs (RR 2.68, 95% CI 1.98–3.63). Subgroup analysis for non-small cell lung cancer (NSCLC) patients also exhibited a greater risk of discontinuation due to TRAEs with Chemo-IO compared to mono-IO (RR 2.93, 95% CI 1.67–5.14). Additional analyses evaluating discontinuation rates due to either treatment emergent adverse events (TEAEs) or AEs regardless of causality (any AEs) consistently revealed an elevated risk associated with Chemo-IO.

**Conclusions:**

Chemo-IO was associated with an elevated risk of treatment discontinuation not only due to TRAEs but also any AEs or TEAEs. Given that the treatment duration can impact clinical outcomes, a subset of patients might benefit more from mono-IO than combination therapy. Further research is imperative to identify and characterize this subset.

**Supplementary Information:**

The online version contains supplementary material available at 10.1186/s12885-024-11897-4.

## Background

Since the regulatory approval of ipilimumab, an anti-CTLA-4 agent, immunotherapies (IO) targeting other immune checkpoints such as programmed cell death protein 1 (PD-1) and its ligand (PD-L1) have emerged [1]. These therapies have revolutionized cancer treatment and have become the standard-of-care for various cancer types [2]. While many predictive biomarkers have been scrutinized to select patients who might benefit from IO, their correlation with treatment responsiveness to IO remains elusive [3–5].

To further enhance the efficacy of IO, combinations with chemotherapy (Chemo-IO) have been introduced, leveraging their synergistic effects [6]. Correspondingly, numerous clinical trials exploring various combination regimens have been conducted. Some of these have already received approval as standard-of-care, while others are still in progress [7].

However, safety profiles significantly impact clinical outcomes, necessitating careful consideration of Chemo-IO’s safety alongside its efficacy. As might be intuitively expected, it is well-recognized that the addition of chemotherapy elevates the severity and frequency of treatment-related adverse events (TRAEs) when compared to IO alone [8, 9]. Nevertheless, the differences in discontinuation rates due to TRAEs between Chemo-IO and IO across various solid tumors have not been fully elucidated.

In this study, we evaluated the safety of Chemo-IO in comparison with IO monotherapy (mono-IO) as a first-line treatment, focusing on TRAEs leading to discontinuation in various types of solid tumors. Given the limited availability of randomized controlled trials (RCTs) directly comparing the safety between Chemo-IO and mono-IO, a network meta-analysis was conducted to establish an indirect comparison between these two groups.

## Methods

The conduct of this meta-analysis adhered to the preferred reporting items for systematic review and meta-analysis (PRISMA) and the PRISMA extension statement for network meta-analysis (Supplementary Table 1) [10].

### Types of adverse events

AEs of any cause (any AEs) are generally defined as any harmful changes in health or unanticipated side-effects experienced by an individual undergoing medical treatment or within a predetermined time period after the completion of treatment. Although any AEs are typically recorded after signing the informed consent form, treatment emergent adverse events (TEAEs) refer to any new events not that were not present prior to treatment initiation or any pre-existing conditions that escalate in severity or frequency post-exposure to the treatment. Therefore, TEAEs are collected after the initiation of the treatment. Moreover, both AEs or TEAEs can either be linked or unlinked to the treatment, while TRAEs are a subcategory of TEAEs deemed as being related to the treatment as determined by the investigator.

### Data sources and search strategies

We systematically searched electronic databases (PubMed, Embase, Cochrane Library, and additional sources) for relevant clinical trials published in English between 01 January 2018 and 10 July 2023. The keywords for the literature search included: “clinical trial”, “phase III”, “first-line”, “immunotherapy”, “nivolumab”, “pembrolizumab”, “atezolizumab”, “PD-1 inhibitor”, “PD-L1 inhibitor”, “programmed cell death-1”, “programmed cell death-ligand 1”, “cemiplimab”, “sintilimab”, “tislelizumab”, “camrelizumab”, “durvalumab”, “avelumab”, “toripalimab”, “ipilimumab”, “sugemalimab”, “tremelimumab”, “serplulimab”, “adebrelimab”, “dostarlimab”, “cosibelimab”, “retifanlimab”, “CTLA-4 inhibitor”, and “cytotoxic T lymphocyte associated antigen-4”. More detailed search strategies are described in Supplementary Table 2.

### Selection criteria

The literature incorporated in this study comprises prospective phase III RCT data pertaining to first-line treatments that utilize either IO agents in at least one arm for patients with solid cancer. To simplify the comparison in this study, we focused on IO agents targeting CTLA-4, PD-1, and PD-L1. Studies providing data on the number of discontinuations of any components of treatment due to TRAEs were included in our analysis. For clinical trials with multiple updated results, we referenced the initial peer-reviewed publication reporting discontinuation rates. This decision was based on the observation that the majority of immune-related adverse events (irAEs) or AEs typically occur within the first 15 weeks of treatment [11, 12].

Our exclusion criteria encompassed: (1) abstracts, posters, conference presentations and unpublished results, (2) inability to obtain full-text or repeated publications, (3) studies with incomplete or ambiguous data, or lack of original extractable data, (4) absence of evaluation indicators, (5) studies combined with other drugs or treatment (e.g., tyrosine kinase inhibitors and radiation therapy), and (6) studies in the adjuvant, neoadjuvant or maintenance treatment setting.

Screening was conducted on titles and abstracts prior to the assessment of full-texts to ascertain eligibility. An online spreadsheet was used to double-check all the included trials to ensure adherence to the inclusion criteria.

### Data extraction

Two investigators, Sangwon Shin and Jimin Moon, independently conducted the database search, and scrutinized the titles, abstracts and full-texts to assess eligibility of studies and extract relevant data. Any disagreements encountered during this process were resolved via discussions with a third investigator, Seunghwan Shin. The extracted data includes elements such as clinical trial names, first author and publication year, indication, treatment group, IO agent name, number of participants included in safety analysis, reported outcomes (types of AEs leading to treatment discontinuation) and median follow-up duration. This information was then collated in an online spreadsheet.

### Risk of bias (RoB) and quality assessment

The quality of methodologies of the included trials was evaluated using the Cochrane Collaboration’s tool (2.0) for assessing the RoB in RCTs [13]. The five bias domains examined were those arising from the randomization process, deviations from intended interventions, missing outcome data, measurement of the outcome, and selection of the reported result. Each of these was evaluated for low risk, high risk or having “some concerns” of bias. Subsequently, an overall RoB for each individual RCT was evaluated.

### Additional analysis of the rate of treatment discontinuation due to either TEAEs or any AEs

In this network meta-analysis, the primary results were the rates of treatment discontinuation due to TRAEs. However, in instances where studies did not report discontinuation rates due to TRAEs, we also gathered data on the rates of discontinuation resulting from either any AEs or TEAEs using the same approach for additional analysis. Given that the incidence of AEs between the time of informed consent signing and the initiation of treatment is expected to be minimal, we conducted an aggregated analysis, combining the discontinuation rates due to either TEAEs or any AEs.

### Statistical analysis

We conducted a network meta-analysis based on the frequentist approach to estimate the relative risks (RRs) for the treatment discontinuation rate due to TRAEs, along with 95% CIs compared to each control group in RCTs. RRs greater than others represented to have more risk of treatment discontinuation. Every treatment arm in each RCT was reclassified into one of five categories: mono-IO (defined as the use of a single IO drug), dual-IO (defined as the use of a combination of IO drugs), Chemo-only (defined as the use of a chemotherapy alone), Chemo-IO (defined as the use of a combination of only one IO drug and other chemotherapy drugs), and Chemo-dual-IO (defined as the use of a combination of two IO drugs and other chemotherapy drugs). In cases where multiple reclassified arms existed in a single RCT, we selected a control arm with an identical chemotherapy regimen to better delineate the impact of IO agents.

The heterogeneity within study designs and inconsistency between designs were assessed using Cochran’s Q statistics and its associated *P*-value. A random effects model was considered when substantial heterogeneity existed. The publication bias of studies was evaluated by Egger’s test with a funnel plot. Analyses were conducted separately for the risks of discontinuation due to TRAEs and those of discontinuation due to either any AEs or TEAEs. As the majority of the first-line trials involving IO were published in NSCLC, we conducted a subgroup analysis specific to NSCLC. A two-sided *P*-value < 0.05 was considered statistically significant. All statistical analyses were performed using R version 4.2.3 with meta and netmeta libraries. In addition, the geometry of the network was demonstrated by using the netgraph function in the netmeta library.

## Results

### Characteristics of the literature

**Fig. 1:**
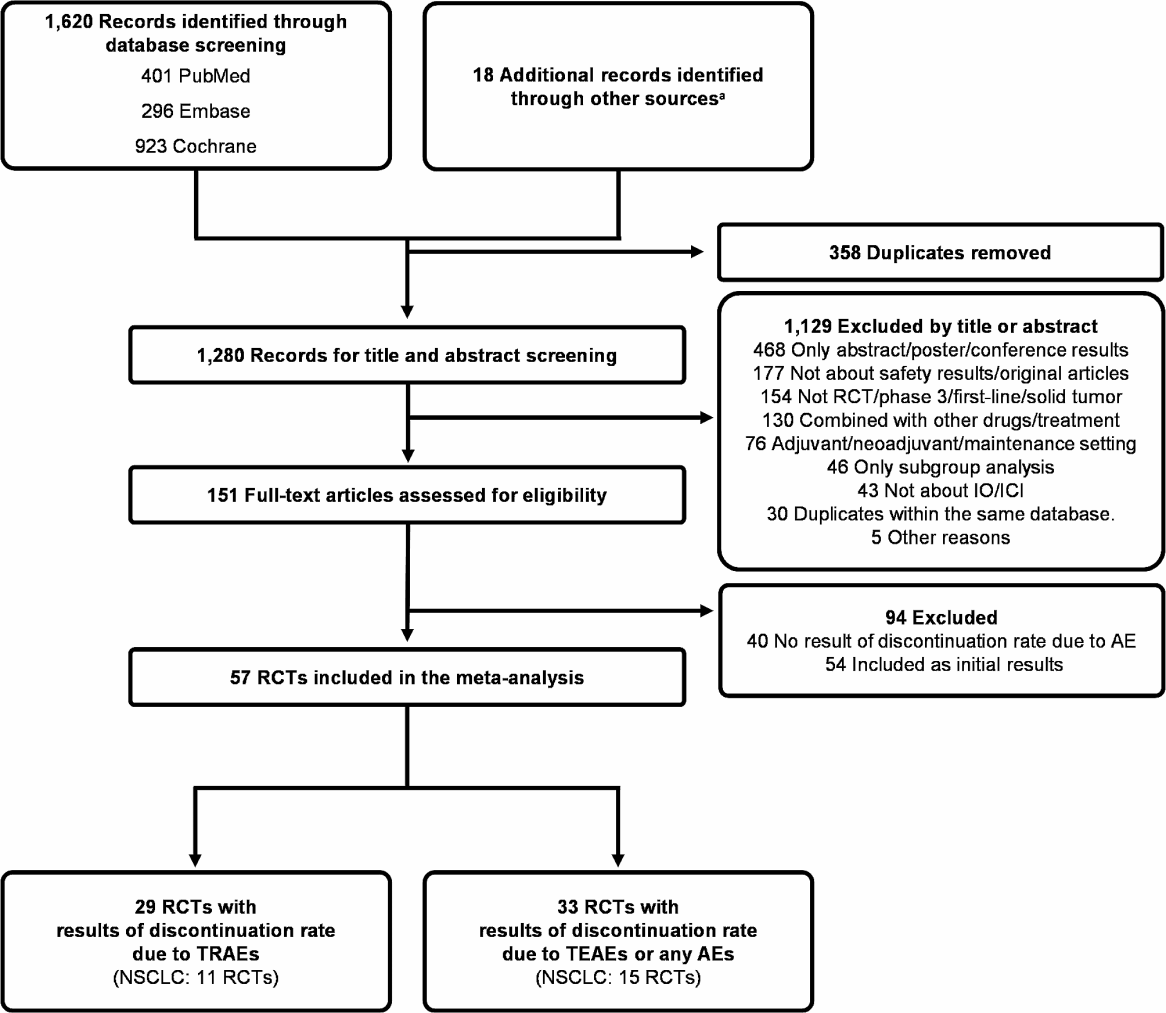
Flowchart of study selection and design ^a^Additional records were identified from NEJM evidence (https://evidence.nejm.org/). AE indicates adverse event; ICI, immune checkpoint inhibitor; IO, immunotherapy; NSCLC, non-small cell lung cancer; RCT, randomized controlled trial; TEAE, treatment emergent adverse event; TRAE, treatment-related adverse event

**Fig. 2:**
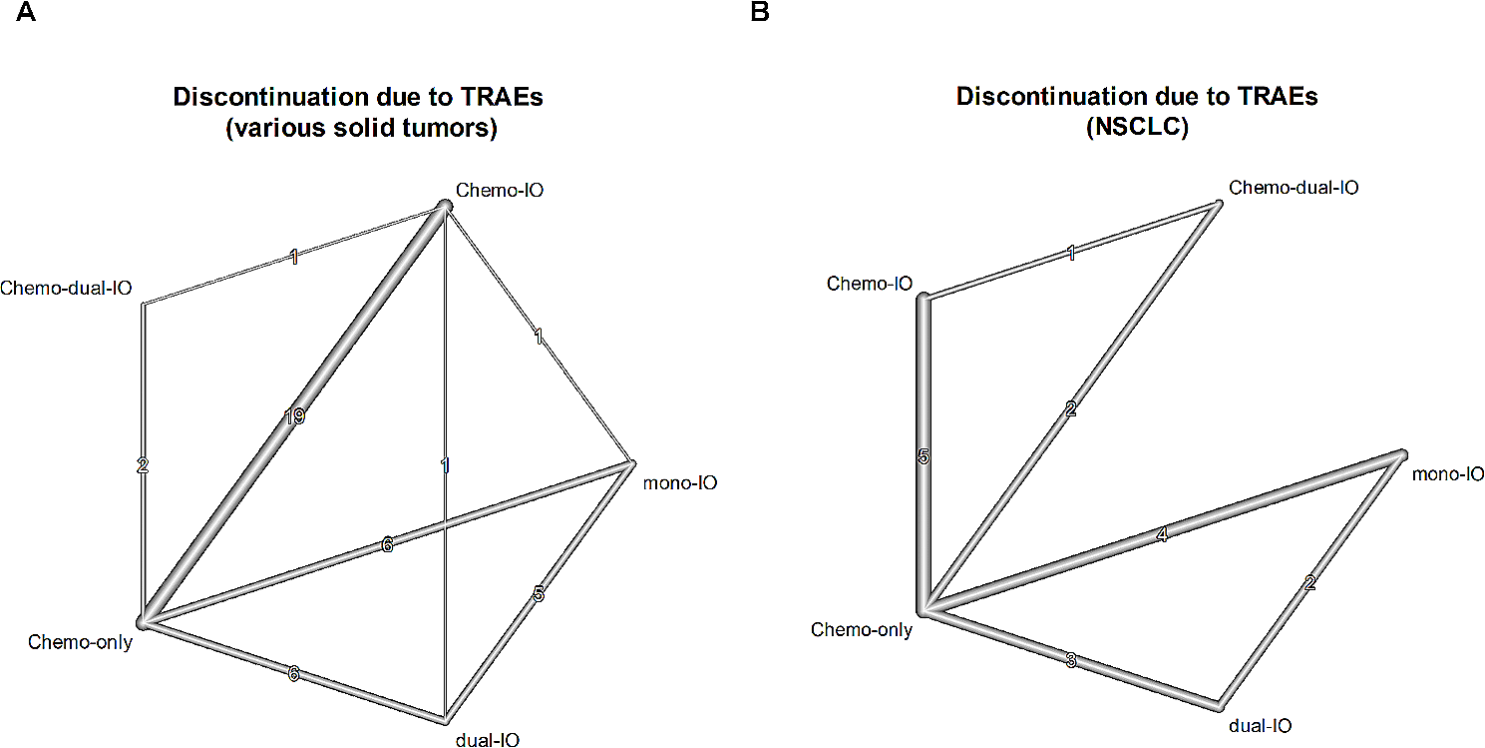
Network plot illustrating comparisons of the risk of discontinuation due to TRAEs among different treatments (**A**) For various cancer types, (**B**) For the NSCLC subgroup

We initially identified a total of 1,620 records from major databases and 18 supplemented publications from NEJM evidence (https://evidence.nejm.org/). After eliminating duplicates and non-relevant literatures through title and abstract screening, we found 151 literatures suitable for full-text review. Of these, 29 RCTs met our inclusion criteria (Fig. 1). Detailed evaluation was conducted on these 29 trials with results of discontinuation rate due to TRAEs (Tables 1 and Fig. 2A). A total of 21,677 patients were included in the safety analysis across various cancer types: NSCLC (11 trials, *n* = 8,442), esophageal squamous cell carcinoma (ESCC) (5 trials, *n* = 3,385), gastrointestinal cancer (GI cancer) (3 trials, *n* = 3,014), biliary tract cancer (BTC) (2 trials, *n* = 1,743), small cell lung cancer (SCLC) (2 trials, *n* = 1,047) and other cancers (6 trials, *n* = 4,046). For the Chemo-IO groups, the following IO agents were used: camrelizumab in 4 arms (*n* = 830), nivolumab in 3 arms (*n* = 1,451), pembrolizumab in 2 arms (*n* = 779), serplulimab in 2 arms (*n* = 771), durvalumab in 2 arms (*n* = 672) and other IO agents across 6 arms (*n* = 1,842). For the mono-IO groups, the following IO agents were used: pembrolizumab in 3 arms (*n* = 1,044), durvalumab in 3 arms (*n* = 1,304), and nivolumab in an arm (*n* = 391).

**Table 1:** Baseline characteristics of studies included in the network meta-analysis

Study (First author, year)	Indication	Treatment group	IO agents	Number of patients in safety analysis	Reported outcomes^a^	Median follow-up duration (months)
ASTRUM-005 [36] (Cheng Y, 2022)	SCLC	Chemo-IO	Serplulimab	389	TRAE	12.3
		Chemo-only	NA	196		12.3
ASTRUM-007 [37] (Song Y, 2023)	ESCC	Chemo-IO	Serplulimab	382	TRAE, TEAE	14.9
		Chemo-only	NA	168		14.9
ATTRACTION-4 [38] (Kang YK, 2022)	GI cancer	Chemo-IO	Nivolumab	359	TRAE	11.6
		Chemo-only	NA	358		11.6
CameL [39] (Zhou C, 2021)	NSCLC	Chemo-IO	Camrelizumab	205	TRAE	11.9
		Chemo-only	NA	207		11.9
CameL-Sq [40] (Ren S, 2022)	NSCLC	Chemo-IO	Camrelizumab	193	TRAE	13.5
		Chemo-only	NA	196		11.6
CAPSTONE-1 [41] (Wang J, 2022)	SCLC	Chemo-IO	Adebrelimab	230	TRAE	13.5
		Chemo-only	NA	232		13.5
CAPTAIN-1st [42] (Yang Y, 2021)	NPC	Chemo-IO	Camrelizumab	134	TRAE, anyAE	15.6
		Chemo-only	NA	129		15.6
Checkmate 227 [43] (Hellmann MD, 2018)	NSCLC	dual-IO	Nivolumab, Ipilimumab	576	TRAE	11.2^b^
		mono-IO	Nivolumab	391		11.2^b^
		Chemo-only	NA	570		11.2^b^
Checkmate 648 [44] (Doki Y, 2022)	ESCC	dual-IO	Nivolumab, Ipilimumab	322	TRAE	13^b^
		Chemo-IO	Nivolumab	310		13^b^
		Chemo-only	NA	304		13^b^
Checkmate 649 [45] (Janjigian YY, 2021)	GI cancer	Chemo-IO	Nivolumab	782	TRAE	13.1
		Chemo-only	NA	767		11.1
Checkmate 743 [46] (Baas P, 2021)	Pleural mesothelioma	dual-IO	Nivolumab, Ipilimumab	300	TRAE	29.7
		Chemo-only	NA	284		29.7
Checkmate 9LA [47] (Paz-Ares L, 2021)	NSCLC	dual-IO	Nivolumab, Ipilimumab	358	TRAE	9.7
		Chemo-only	NA	349		9.7
DANUBE [48] (Powles T, 2020)	MIBC	mono-IO	Durvalumab	345	TRAE, anyAE	41.2
		dual-IO	Durvalumab, Tremelimumab	340		41.2
		Chemo-only	NA	313		41.2
EMPOWER-Lung 3, Part 2 [49] (Gogishvili M, 2022)	NSCLC	Chemo-IO	Cemiplimab	312	TRAE	16.3
		Chemo-only	NA	153		16.7
ESCORT 1st [50] (Luo H, 2021)	ESCC	Chemo-IO	Camrelizumab	298	TRAE	10.8
		Chemo-only	NA	297		10.8
GEMSTONE-302 [51] (Zhou C, 2022)	NSCLC	Chemo-IO	Sugemalimab	320	TRAE	17.8
		Chemo-only	NA	159		17.8
HIMALAYA [52] (Abou-Alfa GK, 2022)	HCC	mono-IO	Durvalumab	388	TRAE	32.6
		dual-IO^c^	Durvalumab, Tremelimumab	540		33.2
JAVELIN Ovarian 100 [53] (Monk BJ, 2021)	Ovarian cancer	Chemo-IO	Avelumab	329	TRAE	12.6
		Chemo	Chemo	334		11.8
KESTREL [54] (Psyrri A, 2023)	HNSCC	mono-IO	Durvalumab	202	TRAE	NA
		dual-IO	Durvalumab. Tremelimumab	408		NA
KEYNOTE-024 [55] (Reck M, 2016)	NSCLC	mono-IO	Pembrolizumab	154	TRAE	11.2
		Chemo-only	NA	150		11.2
KEYNOTE-042 [56] (Mok TSK, 2019)	NSCLC	mono-IO	Pembrolizumab	636	TRAE	12.8
		Chemo-only	NA	615		12.8
KEYNOTE-062 [57] (Shitara K, 2020)	GI cancer	mono-IO	Pembrolizumab	254	TRAE	29.4
		Chemo-IO	Pembrolizumab	250		29.4
		Chemo-only	NA	244		29.4
KEYNOTE-966 [58] (Kelley RK, 2023)	BTC	Chemo-IO	Pembrolizumab	529	TRAE	25.6
		Chemo-only	NA	534		25.6
MYSTIC [59] (Rizvi NA, 2020)	NSCLC	mono-IO	Durvalumab	369	TRAE	30.2
		dual-IO	Durvalumab, Tremelimumab	371		30.2
		Chemo-only	NA	352		30.2
NEPTUNE [60] (de Castro G, 2023)	NSCLC	dual-IO	Durvalumab, Tremelimumab	410	TRAE	32.9
		Chemo-only	NA	399		32.9
ORIENT-15 [61] (Lu Z, 2022)	ESCC	Chemo-IO	Sintilimab	327	TRAE, TEAE	16.0
		Chemo-only	NA	332		16.9
Poseidon [62] (Johnson ML, 2023)	NSCLC	Chemo-dual-IO	Durvalumab, Tremelimumab	330	TRAE	15.5
		Chemo-IO	Durvalumab	334		15.5
		Chemo-only	NA	333		15.5
RATIONALE-306 [63] (Xu J, 2023)	ESCC	Chemo-IO	Tislelizumab	324	TRAE	16.3
		Chemo-only	NA	321		9.8
TOPAZ-1 [64] (Oh DY, 2022)	BTC	Chemo-IO	Durvalumab	338	TRAE, anyAE	16.8
		Chemo-only	NA	342		15.9

^a^Types of AEs leading to treatment discontinuation

^b^Only reported minimum duration of follow-up

^c^Two arms using the same ICI agents with different strategies combined

Abbreviations: AE, adverse event; BTC, biliary tract cancer; Chemo-dual-IO, dual-immunotherapy combined with chemotherapy; Chemo-IO, mono-immunotherapy combined with chemotherapy; Chemo-only, chemotherapy alone; dual-IO, dual-immunotherapy; ESCC, esophageal squamous cell carcinoma; GI, gastrointestinal; HCC, hepatocellular carcinoma; HNSCC, head and neck squamous cell carcinoma; IO, immunotherapy; MIBC, muscle invasive bladder cancer; mono-IO, mono-immunotherapy; NA, not applicable; NPC, nasopharyngeal carcinoma; NSCLC, non-small cell lung cancer; SCLC, small cell lung cancer; TEAE, treatment emergent adverse event; TRAE, treatment-related adverse event;

The width of lines is proportional to the number of trials that compare directly in the same trial. Chemo-dual-IO indicates dual-immunotherapy combined with chemotherapy; Chemo-IO, mono-immunotherapy combined with chemotherapy; Chemo-only, chemotherapy alone; dual-IO, dual-immunotherapy; mono-IO, mono-immunotherapy; NSCLC, non-small cell lung cancer; TRAE, treatment-related adverse event.

### Assessment of RoB and quality assessment

The summary of the RoB assessment for the included studies is presented in Supplementary Fig. 1. Given that this network meta-analysis focuses on treatment discontinuation, deviations from intended interventions inevitably raise some concerns or result in a high risk. Furthermore, due to the variation in median follow-up durations across the RCTs, the selection of the reported result domain was unavoidably rated as high risk. As a result, all RCTs were found to have a high overall RoB. Nevertheless, based on the funnel plot and Egger’s test, there is no definite evidence of publication bias concerning the discontinuation rate due to TRAEs (see Supplementary Fig. 2).

### Comparison of the rate of treatment discontinuation due to TRAEs across all solid cancers

Substantial heterogeneity within study designs was observed among the results of each study (Q = 50.69, *P* < 0.01), leading to the application of a random effects model. Additionally, the Q statistic for testing inconsistency between designs, when using a full design-by-treatment interaction random effect model, was no longer significant (changing from Q = 31.93, *P* < 0.01 to Q = 13.69, *P* = 0.13).

In direct comparisons with Chemo-only, Chemo-IO exhibited a substantially higher rate of treatment discontinuation due to TRAEs (RR 1.60, 95% CI 1.36–1.88), whereas mono-IO showed a reduced rate of discontinuation due to TRAEs (RR 0.67, 95% CI 0.50–0.90). Additionally, a significant, albeit small, difference was observed between dual-IO and Chemo-only (RR 1.35, 95% CI 1.04–1.74). Dual-IO exhibited nearly twice the rate of discontinuation compared to mono-IO (RR 2.09, 95% CI 1.51–2.91). Outcomes from direct comparisons with only a limited number of trials, such as between Chemo-IO and mono-IO, were considered less reliable.

**Fig. 3:**
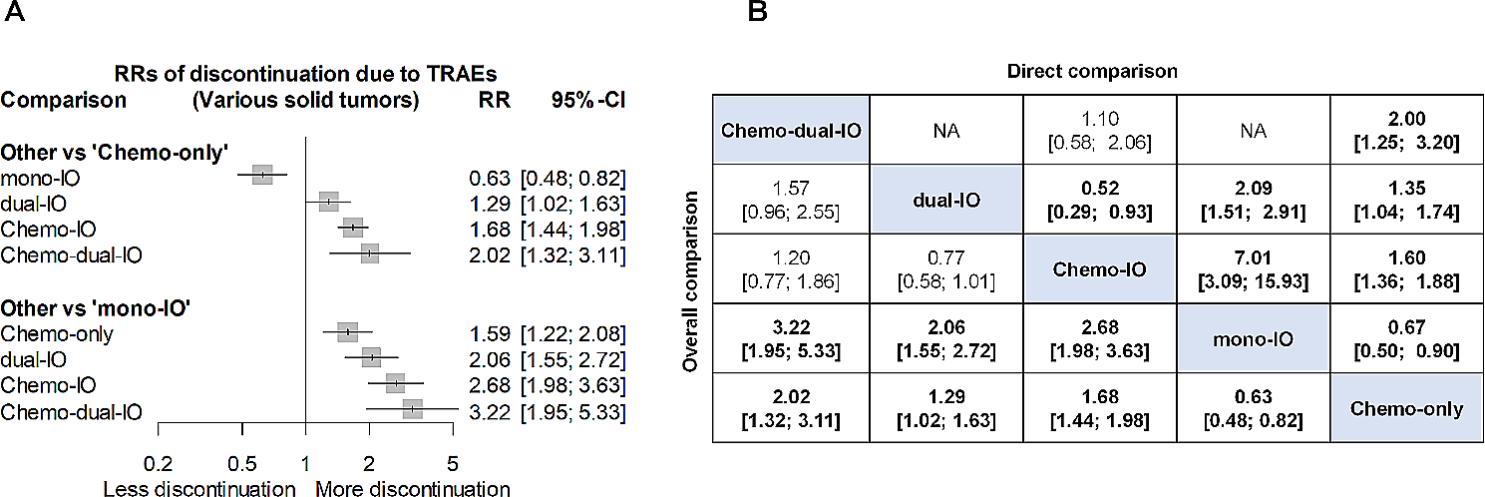
Relative risks (RRs) of discontinuation due to TRAEs across various solid tumors (**A**) Forest plot of RR of treatment discontinuation due to TRAEs. (**B**) Direct and overall comparisons of discontinuation due to TRAEs. Each cell contains pooled RR with 95% CI. An RR greater than 1 means that upper-row treatment has a greater risk of treatment discontinuation. The overall comparison outcomes, which combine direct and indirect comparison results from the network meta-analysis are displayed in the left lowerhalf, while results from pairwise meta-analysis are shown in the right upper half, if available. Significant results are in bold. Chemo-dual-IO indicates dual-immunotherapy combined with chemotherapy; Chemo-IO, mono-immunotherapy combined with chemotherapy; Chemo-only, chemotherapy alone; CI, confidence interval; dual-IO, dual-immunotherapy; mono-IO, mono-immunotherapy; NA, not available; RR, relative risk; TRAE, treatment-related adverse event.

In the overall comparison, which combines the outcomes of direct comparison with indirect comparisons from the network meta-analysis, Chemo-IO exhibited a notably higher rate of discontinuation due to TRAEs compared to mono-IO (RR 2.68, 95% CI 1.98–3.63). Furthermore, when compared to Chemo-only, Chemo-IO presented an increased rate of discontinuation (RR 1.68, 95% CI 1.44–1.98), while mono-IO displayed a decreased rate (RR 0.63, 95% CI 0.48–0.82). A comprehensive summary of all RRs for discontinuation due to TRAEs across five treatment types can be found in Fig. 3.

### Subgroup analysis of the rate of treatment discontinuation due to TRAEs in NSCLC

Out of 29 RCTs reporting on the rate of treatment discontinuation due to TRAEs, 11 RCTs focused on NSCLC (Fig. 2B). In line with the analysis conducted across various cancer types, a random effects model was applied due to substantial heterogeneity with designs (Q = 17.54, *P* < 0.01). Additionally, after applying a random effects model, the significance indicating inconsistency between designs was no longer positive (changing from Q = 8.56, *P* = 0.07 to Q = 2.82, *P* = 0.59).

**Fig. 4:**
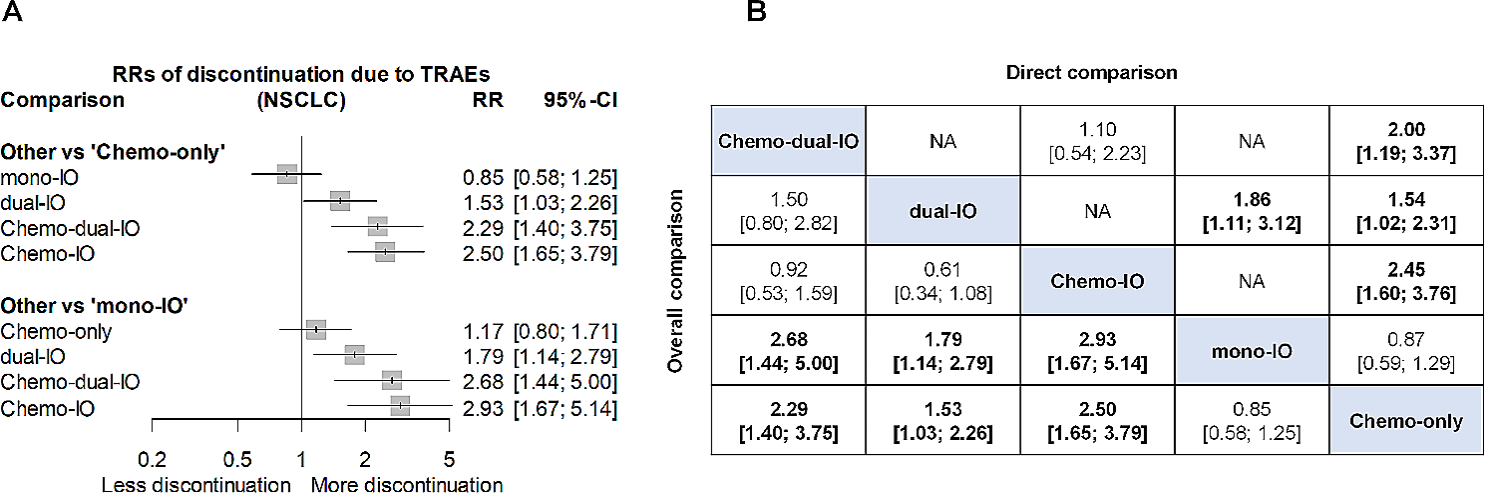
Relative risks (RRs) of discontinuation due to TRAEs in NSCLC. (**A**) Forest plot of RR of treatment discontinuation due to TRAEs. (**B**) Direct and overall comparisons of discontinuation due to TRAEs. Each cell contains pooled RR with 95% CI. An RR greater than 1 means that upper-row treatment has a greater risk of treatment discontinuation. The overall comparison outcomes, which combine direct and indirect comparison results from the network meta-analysis are displayed in the left lower half, while results from pairwise meta-analysis are shown in the right upper half, if available. Significant results are in bold. Chemo-dual-IO indicates dual-immunotherapy combined with chemotherapy; Chemo-IO, mono-immunotherapy combined with chemotherapy; Chemo-only, chemotherapy alone; CI, confidence interval; dual-IO, dual-immunotherapy; mono-IO, mono-immunotherapy; NA, not available; NSCLC, non-small cell lung cancer; RR, relative risk; TRAE, treatment-related adverse event

When directly compared to Chemo-only, Chemo-IO was associated with a significantly higher rate of discontinuation due to TRAEs (RR 2.45, 95% CI 1.60–3.76), On the other hand, mono-IO presented a decreased rate of discontinuation due to TRAEs, although this was not statistically significant (RR 0.87, 95% CI 0.59–1.29). Through indirect comparison, we observed that Chemo-IO had a considerably higher rate of discontinuation owing to TRAEs when compared to mono-IO (RR 2.93, 95% CI 1.67–5.14). All RRs for discontinuation due to TRAEs are summarized in Fig. 4.

### Comparison of the rate of treatment discontinuation due to any AEs or TEAEs

For the studies not reporting discontinuation rates due to TRAEs, we collected the discontinuation rates associated with either any AEs or TEAEs. These data were sourced from 33 RCTs, encompassing a safety analysis of 18,482 patients (Fig. 1 and Supplementary Table 3). Using these data, we conducted an aggregate analysis integrating the discontinuation rates stemming from either any AEs or TEAEs (Supplementary Fig. 3).

Given that significant within-design heterogeneity was evident (Q = 96.47, *P* < 0.01), a random effects model was adopted. Under this model, the inconsistency between designs continued to be non-significant, as indicated by the change in Q value (from Q = 8.66, *P* = 0.12 to Q = 2.70, *P* = 0.75). Overall, when combining direct and indirect comparison results, Chemo-IO demonstrated a significantly higher rate of discontinuation due to any AEs/TEAEs compared to mono-IO (RR 2.48, 95% CI 1.93–3.17) (Supplementary Fig. 4). Similarly, for NSCLC patients, Chemo-IO showed a markedly elevated rate of discontinuation due to any AEs/TEAEs in comparison to mono-IO, as deduced from the indirect comparisons of Chemo-IO or mono-IO against Chemo-only (RR 2.09, 95% CI 1.29–3.39) (Supplementary Fig. 5).

## Discussion

In our network meta-analysis evaluating first-line treatments for diverse solid tumors, we found that patients treated with Chemo-IO experienced a higher rate of treatment discontinuation due to TRAEs than those receiving mono-IO.

The advent of immunotherapy has ushered in a new era in cancer treatment. Starting with mono-IO, the field has since evolved to incorporate more complex regimens, such as IO-IO combinations and Chemo-IO, which have shown improved survival outcomes over Chemo-only or mono-IO [2, 6, 7, 14]. These advancements have led to approvals from the U.S. Food and Drug Administration (FDA) and the European Medicines Agency (EMA) for various cancer treatment settings [7, 8, 15, 16].

However, due to the additive nature of adverse effects, Chemo-IO is generally associated with a higher incidence of TRAEs compared to mono-IO [8, 9]. Consistent with these findings, our study observed a significantly increased rate of TRAE-induced discontinuation in the Chemo-IO group compared to the mono-IO group across various cancer types. Similarly, among NSCLC patients, Chemo-IO also demonstrated an elevated rate of discontinuation due to TRAEs compared with mono-IO.

The impact of treatment discontinuation due to AEs on clinical outcomes remains a subject of debate. Some studies have observed significantly poorer outcomes in the discontinuation group compared to the non-discontinuation group [17–19], while some studies have found no significant difference in clinical outcomes between groups that discontinued treatment due to AEs and those that did not [20–22], Although treatment discontinuation may occur because of AEs, several factors—including the timing of AEs, the patient’s response prior to discontinuation, the severity of the AEs or other variables—could be associated with clinical outcomes after discontinuation [17, 19, 23]. For instance, one study found that early AEs, occurring before 12 weeks and often leading to discontinuation, were associated with worse survival outcomes than later AEs [17]. Another study demonstrated that patients achieving complete response (CR) or partial response (PR) before the occurrence of the first irAE showed no difference in clinical outcomes between those who discontinued treatment and those who did not or those who resumed treatment. Conversely, patients who did not achieve CR or PR before the first irAE experienced significantly poorer outcomes in the discontinuation group compared to the non-discontinuation or re-treatment group [19]. Furthermore, mild irAEs might be indicative of a favorable response to immunotherapy, while severe irAEs (grade ≥ 3), often life-threatening or leading to treatment discontinuation, may not be indicative of favorable clinical outcomes [23]. TRAEs that occur during IO or Chemo-IO treatment could be linked to life-threatening conditions or mortality, leading physicians to generally consider them as potentially detrimental. In this study, we primarily suggested that IO-chemotherapy combination treatments are associated with a high rate of discontinuation. And there is a need for additional studies to investigate the association between AE- or TRAE-induced discontinuation and clinical outcomes.

Furthermore, the optimal duration for IO treatment continues to be a topic of ongoing debate, several trials, including KEYNOTE-189 [24], KEYNOTE-010 [25], and CheckMate-153 [26], suggest the possibility of poor survival outcomes or disease progression after discontinuing IO treatment following one or two years of administration [27]. Limited research has managed to follow patients who discontinue treatment due to AEs. However, one retrospective study that examined the clinical outcomes of patients who discontinued IO treatment due to immune-related AEs found that 20% of patients experienced disease progression within six months of discontinuation and 10% of patients died [28]. This evidence further emphasizes the potential impact of treatment discontinuation on patient outcomes.

In susceptible populations, such as elderly patients or those with a poor Eastern Cooperative Oncology Group performance status (ECOG PS), the administration of Chemo-IO could potentially be associated with a poorer prognosis due to an increased incidence of severe TRAEs and subsequent treatment discontinuation owing to these AEs [29]. Moreover, even with Chemo-only regimens, certain patient subgroups such as those of advanced age, poor performance status, individuals with anemia, impaired renal function, hearing impairment, or history of falls, are known to have an increased risk of toxicity [30]. This heightened toxicity could potentially lead to treatment discontinuation in these subgroups. However, enrolling these specific subgroups in oncological trials poses challenges, leaving unresolved questions and concerns about the extent of harm caused by treatment discontinuation in these populations.

For high-risk patients predicted to cease treatment due to TRAEs, a treatment strategy focusing on mono-IO might offer a lower discontinuation rate, thus improving treatment continuity. Furthermore, considering that several studies have reported comparable effects of IO to Chemo-IO in certain subgroups, such as patients aged 75 or over, or those who have received prior treatments [16, 31], there is a pressing need for further research into potential biomarkers or clinical factors that can aid in identifying patients who would benefit sufficiently from mono-IO instead of Chemo-IO, especially since these populations may experience higher rates of treatment discontinuation with Chemo-IO.

Several predictive biomarkers, including PD-L1, tumor mutation burden (TMB), and tumor-infiltrating lymphocytes (TILs), have been proposed to predict the efficacy of IO, but their roles remain inconclusive [3, 4, 8, 32]. Consequently, novel approaches are being explored to improve prediction accuracy, including the application of artificial intelligence (AI). Some studies have even reported that AI-assisted methods, such as the evaluation of pretreatment contrast-enhanced CT images, PD-L1 expression, or the spatial analysis of TIL, can yield better predictions of survival outcomes in NSCLC patients undergoing IO treatment [33–35]. Advancing this line of research might not only refine predictions of IO efficacy but also help in anticipating and managing treatment discontinuation, thus facilitating more personalized treatment strategies and potentially improving patient outcomes.

### Limitations

While this meta-analysis provides valuable insights, it does have certain limitations. First, the potential for bias due to confounding effects could vary based on the types of immunotherapies used across the different regimens and the diversity in types of solid tumors. However, due to the lack of sufficient data from the RCTs, we did not differentiate among these agents in our analysis. Second, our study is primarily based on published literature and clinical trial results, which could potentially lead to publication bias, as studies with negative results are less likely to be published. Third, the duration of follow-up in each RCT differed, which could have potentially influenced the uncertainty of our pooled results. However, it is important to note that the majority of irAEs or AEs typically occur within the first 15 weeks of treatment. Therefore, the impact of varying follow-up durations on our findings is likely to be minimal. Last, we did not have access to individual patient data, which restricted us from performing subgroup analysis based on various clinical factors, including age, gender, as well as types and grades of TRAEs.

## Conclusions

While the efficacy of Chemo-IO is generally acknowledged as superior to that of mono-IO, our study has supported that Chemo-IO is linked with a significantly higher rate of treatment discontinuation due to TRAEs. This finding underscores the importance of personalized treatment approaches. For certain patients, particularly those at a heightened risk of TRAEs, mono-IO might be the preferable option due to its lower discontinuation rate. Conversely, for other patients, the potential superior efficacy of Chemo-IO could outweigh the higher risk of discontinuation. Future research should focus on the identification of novel biomarkers and patient characteristics, which could guide the selection of the most appropriate treatment modality, taking into account both efficacy and the likelihood of treatment discontinuation. By customizing treatment strategies to align with individual patient profiles, we aim to enhance therapeutic efficacy while minimizing the risk of premature treatment discontinuation. This balanced approach emphasizes the need for a more nuanced understanding of individual patient responses to different cancer therapies, guiding the evolution of oncology towards a more adaptable and patient-focused discipline.

### Electronic supplementary material

Below is the link to the electronic supplementary material.

Supplementary Material 1

## Data Availability

The datasets used and/or analyzed during the current study are available from the corresponding author on reasonable request. Supplementary information is also accessible in the supplementary materials.

## Abbreviations

AI: Artificial intelligence
AE: Adverse event
any AE: AEs regardless of causality
BTC: Biliary tract cancer
Chemo-dual-IO: The use of a combination of two IO drugs and other chemotherapy drugs
Chemo-IO: The use of a combination of only one IO drug and other chemotherapy drugs
Chemo-only: The use of a chemotherapy alone
CI: Confidence interval
dual-IO: The use of a combination of IO drugs
ECOG PS: Eastern Cooperative Oncology Group performance status
EMA: European Medicines Agency
ESCC: Esophageal squamous cell carcinoma
FDA: Food and Drug Administration
GI cancer: Gastrointestinal cancer
HCC: Hepatocellular carcinoma
HNSCC: Head and neck squamous cell carcinoma
IO: Immunotherapy
irAE: Immune-related adverse event
MIBC: Muscle invasive bladder cancer
mono-IO: The use of a single IO drug
NA: Not applicable
NPC: Nasopharyngeal carcinoma
NSCLC: Non-small cell lung cancer
PD-1: Programmed cell death protein 1
PD-L1: Programmed cell death ligand 1
PRISMA: Preferred reporting items for systematic review and meta-analysis
RCT: Randomized controlled trial
RoB: Risk of bias
RR: Relative risk
SCLC: Small cell lung cancer
TEAE: Treatment emergent adverse event
TIL: Tumor-infiltrating lymphocyte
TMB: Tumor mutation burden
TRAE: Treatment related adverse event
