# Supplementary material for: Discontinuation risk from adverse events: immunotherapy alone vs. combined with chemotherapy: a systematic review and network meta-analysis
Source: BMC Cancer. 2024 Jan 30;24:152. doi: 10.1186/s12885-024-11897-4 (PMC10825980; doi:10.1186/s12885-024-11897-4)
Supplement: Supplementary file 1 — Supplementary Material 1 [file 12885_2024_11897_MOESM1_ESM.docx]

# Supplemental Online Content

**Supplementary Table 1. PRISMA extension checklist for network meta-analysis**

**Supplementary Table 2. Search Strategy**

**Supplementary Table 3. Baseline Characteristics of studies included in the additional analysis of the risk of treatment discontinuation due to any AEs or TEAEs**

**Supplementary Figure 1. Assessment of Risk of Bias**

**Supplementary Figure 2. Assessment of Publication Bias**

**Supplementary Figure 3. Network plot illustrating comparisons of relative risks (RRs) of discontinuation due to any AEs or TEAEs among different treatment regimens**

**Supplementary Figure 4. Relative risks (RRs) of discontinuation due to any AEs or TEAEs across various solid tumors**

**Supplementary Figure 5. Relative risks (RRs) of discontinuation due to any AEs or TEAEs in NSCLC**

**Supplementary References**

## Supplementary Table 1. PRISMA extension checklist for network meta-analysis

| **Section/Topic** | **Item #** | **Checklist Item** | **Reported on Page #** |
| --- | --- | --- | --- |
| **TITLE** | | | |
| Title | 1 | Identify the report as a systematic review *incorporating a network meta-analysis (or related form of*  *meta-analysis).* | *#1* |
| **ABSTRACT** | | | |
| Structured summary | 2 | Provide a structured summary including, as applicable:  **Background:** main objectives  **Methods:** data sources; study eligibility criteria, participants, and interventions; study appraisal; and *synthesis methods, such as network meta-analysis.*  **Results:** number of studies and participants identified; summary estimates with corresponding confidence/credible intervals; *treatment rankings may also be discussed. Authors may choose to summarize pairwise comparisons against a chosen treatment included in their analyses for brevity.*  **Discussion/Conclusions:** limitations; conclusions and implications of findings.  **Other:** primary source of funding; systematic review registration number with registry name. | *#2-4* |
| **INTRODUCTION** | | | |
| Rationale | 3 | Describe the rationale for the review in the context of what is already known*, including mention of why a network meta-analysis has been conducted.* | *#5-6* |
| Objectives | 4 | Provide an explicit statement of questions being addressed, with reference to participants, interventions, comparisons, outcomes, and study design (PICOS). | *#5-6* |
| **METHODS** | | | |
| Protocol and registration | 5 | Indicate whether a review protocol exists and if and where it can be accessed (e.g., Web address); and, if available, provide registration information, including registration number. | *Not registered* |
| Eligibility criteria | 6 | Specify study characteristics (e.g., PICOS, length of follow-up) and report characteristics (e.g., years considered, language, publication status) used as criteria for eligibility, giving rationale. *Clearly describe eligible treatments included in the treatment network, and note whether any have been clustered or merged into the same node (with justification).* | *#6-7* |
| Information sources | 7 | Describe all information sources (e.g., databases with dates of coverage, contact with study authors to identify additional studies) in the search and date last searched. | *#6* |
| Search | 8 | Present full electronic search strategy for at least one database, including any limits used, such that it could be repeated. | *STable 2* |
| Study selection | 9 | State the process for selecting studies (i.e., screening, eligibility, included in systematic review, and, if applicable, included in the meta-analysis). | *#6-7, Figure 1* |
| Data collection process | 10 | Describe method of data extraction from reports (e.g., piloted forms, independently, in duplicate) and any processes for obtaining and confirming data from investigators. | *#7* |
| Data items | 11 | List and define all variables for which data were sought (e.g., PICOS, funding sources) and any assumptions and simplifications made. | *#7* |
| Geometry of the network | S1 | Describe methods used to explore the geometry of the treatment network under study and potential biases related to it. This should include how the evidence base has been graphically summarized for presentation, and what characteristics were compiled and used to describe the evidence base to readers. | *#8* |
| Risk of bias within individual studies | 12 | Describe methods used for assessing risk of bias of individual studies (including specification of whether this was done at the study or outcome level), and how this information is to be used in any data synthesis. | *#7* |
| Summary measures | 13 | State the principal summary measures (e.g., risk ratio, difference in means). *Also describe the use of additional summary measures assessed, such as treatment rankings and surface under the cumulative ranking curve (SUCRA) values, as well as modified approaches used to present summary findings from meta-analyses.* | *#8* |
| Planned methods of analysis | 14 | Describe the methods of handling data and combining results of studies for each network meta-analysis. This should include, but not be limited to:   - *Handling of multi-arm trials;* - *Selection of variance structure;* - *Selection of prior distributions in Bayesian analyses; and* - *Assessment of model fit.* | *#8* |
| Assessment of Inconsistency | S2 | Describe the statistical methods used to evaluate the agreement of direct and indirect evidence in the treatment network(s) studied. Describe efforts taken to address its presence when found. | *#8* |
| Risk of bias across studies | 15 | Specify any assessment of risk of bias that may affect the cumulative evidence (e.g., publication bias, selective reporting within studies). | *#8* |
| Additional analyses | 16 | Describe methods of additional analyses if done, indicating which were pre-specified. This may include, but not be limited to, the following:   - Sensitivity or subgroup analyses; - Meta-regression analyses; - *Alternative formulations of the treatment network; and* - *Use of alternative prior distributions for Bayesian analyses (if applicable).* | *#8* |
| **RESULTS†** | | | |
| Study selection | 17 | Give numbers of studies screened, assessed for eligibility, and included in the review, with reasons for exclusions at each stage, ideally with a flow diagram. | *#9* |
| Presentation of network structure | S3 | Provide a network graph of the included studies to enable visualization of the geometry of the treatment network. | *Figure 2* |
| Summary of network geometry | S4 | Provide a brief overview of characteristics of the treatment network. This may include commentary on the abundance of trials and randomized patients for the different interventions and pairwise comparisons in the network, gaps of evidence in the treatment network, and potential biases reflected by the network structure. | *#9* |
| Study characteristics | 18 | For each study, present characteristics for which data were extracted (e.g., study size, PICOS, follow-up period) and provide the citations. | *Table 1* |
| Risk of bias within studies | 19 | Present data on risk of bias of each study and, if available, any outcome level assessment. | *SFigure 1* |
| Results of individual studies | 20 | For all outcomes considered (benefits or harms), present, for each study: 1) simple summary data for each intervention group, and 2) effect estimates and confidence intervals. *Modified approaches may be needed to deal with information from larger networks.* | *#9* |
| Synthesis of results | 21 | Present results of each meta-analysis done, including confidence/credible intervals. *In larger networks, authors may focus on comparisons versus a particular comparator (e.g., placebo or standard care), with full findings presented in an appendix. League tables and forest plots may be considered to summarize pairwise comparisons.* If additional summary measures were explored (such as treatment rankings), these should also be presented. | *#9-11* |
| Exploration for inconsistency | S5 | Describe results from investigations of inconsistency. This may include such information as measures of model fit to compare consistency and inconsistency models, *P* values from statistical tests, or summary of inconsistency estimates from different parts of the treatment network. | *#10-11* |
| Risk of bias across studies | 22 | Present results of any assessment of risk of bias across studies for the evidence base being studied. | *#9-11* |
| Results of additional analyses | 23 | Give results of additional analyses, if done (e.g., sensitivity or subgroup analyses, meta-regression analyses*, alternative network geometries studied, alternative choice of prior distributions for Bayesian analyses,* and so forth). | *#10-11* |
| **DISCUSSION** | | | |
| Summary of evidence | 24 | Summarize the main findings, including the strength of evidence for each main outcome; consider their relevance to key groups (e.g., healthcare providers, users, and policy-makers). | *#12* |
| Limitations | 25 | Discuss limitations at study and outcome level (e.g., risk of bias), and at review level (e.g., incomplete retrieval of identified research, reporting bias). *Comment on the validity of the assumptions, such as transitivity and consistency. Comment on any concerns regarding network geometry (e.g., avoidance of certain comparisons).* | *#13* |
| Conclusions | 26 | Provide a general interpretation of the results in the context of other evidence, and implications for future research. | *#14* |
| **FUNDING** | | | |
| Funding | 27 | Describe sources of funding for the systematic review and other support (e.g., supply of data); role of funders for the systematic review. This should also include information regarding whether funding has been received from manufacturers of treatments in the network and/or whether some of the authors are content experts with professional conflicts of interest that could affect use of treatments in the network. | *unfunded research* |

PICOS = population, intervention, comparators, outcomes, study design.

* Text in italics indicates wording specific to reporting of network meta-analyses that has been added to guidance from the PRISMA statement.

† Authors may wish to plan for use of appendices to present all relevant information in full detail for items in this section.

## Supplementary Table 2. Search Strategy (1,620 results)

| **PubMed**: 401 results |
| --- |
| (Clinical Trial[Filter]) AND ((Neoplasm[MeSH Terms]) OR (cancer) OR (carcinoma)) AND ((immunotherapy[MeSH Terms]) OR (chemotherapy)) AND ((first-line) OR (first line) OR (first) OR (untreated)) AND ((immune checkpoint inhibitor) OR (nivolumab) OR (pembrolizumab) OR (atezolizumab) OR (PD-1 inhibitor) OR (PD-L1 inhibitor) OR (programmed cell death-1) OR (programmed cell death-ligand 1) OR (cemiplimab) OR (sintilimab) OR (tislelizumab) OR (camrelizumab) OR (durvalumab) OR (avelumab) OR (toripalimab) OR (ipilimumab) OR (sugemalimab) OR (tremelimumab) OR (serplulimab) OR (adebrelimab) OR (dostarlimab) OR (cosibelimab) OR (retifanlimab) OR (CTLA-4 inhibitor) OR (cytotoxic T lymphocyte associated antigen-4)) AND ((Clinical Trial, Phase III[pt]) OR (Phase III) OR (Randomized Controlled Trial[pt]) OR (KEYNOTE) OR (CheckMate) OR (RATIONALE)) AND (("2018/01/01"[Date - Publication] : "3000"[Date - Publication])) NOT ((Phase 1[Title]) OR (Phase I[Title]) OR (Phase 2[Title]) OR (Phase II[Title]) OR (Phase 1b[Title]) OR (Phase Ib[Title]) OR (Phase 0[Title]) OR (Phase 2b[Title]) OR (Phase IIb[Title])) NOT (cost[Title]) |
| **Embase**: 296 results |
| ('neoplasm'/exp OR 'cancer':ab,ti OR 'carcinoma':ab,ti OR 'tumor':ab,ti) AND ('phase 3 clinical trial'/exp OR 'phase 3':ab,ti OR 'phase iii':ab,ti OR 'keynote':ab,ti OR 'checkmate':ab,ti OR 'rationale':ab,ti) AND ('controlled study'/de OR 'major clinical study'/de OR 'phase 3 clinical trial'/de OR 'randomized controlled trial'/de) AND ('cancer immunotherapy'/exp OR 'cancer chemotherapy'/exp OR 'immunotherapy':ab,ti OR 'chemotherapy':ab,ti OR 'platinum':ab,ti OR 'pemetrexed':ab,ti) AND ('first-line treatment'/exp OR 'first line therapy':ab,ti OR 'first':ab,ti OR 'untreated':ab,ti) AND ('immune checkpoint inhibitor':ab,ti OR 'nivolumab':ab,ti OR 'pembrolizumab':ab,ti OR 'atezolizumab':ab,ti OR 'pd-1 inhibitor':ab,ti OR 'pd-l1 inhibitor':ab,ti OR 'programmed cell death-1':ab,ti OR 'programmed cell death-ligand 1':ab,ti OR 'cemiplimab':ab,ti OR 'sintilimab':ab,ti OR 'tislelizumab':ab,ti OR 'camrelizumab':ab,ti OR 'durvalumab':ab,ti OR 'avelumab':ab,ti OR 'toripalimab':ab,ti OR 'ipilimumab':ab,ti OR 'sugemalimab':ab,ti OR 'tremelimumab':ab,ti OR 'serplulimab':ab,ti OR 'adebrelimab':ab,ti OR 'dostarlimab':ab,ti OR 'cosibelimab':ab,ti OR 'retifanlimab':ab,ti OR 'ctla-4 inhibitor':ab,ti OR 'cytotoxic t lymphocyte associated antigen-4':ab,ti) AND [2018-2023]/py NOT ('phase 1':ab,ti OR 'phase i':ab,ti OR 'phase ii':ab,ti OR 'phase 2':ab,ti OR 'phase 1b':ab,ti OR 'phase 2b':ab,ti OR 'real-world':ab,ti OR 'phase 3b/4':ab,ti OR 'cost':ab,ti OR 'food':ab,ti OR 'pooled':ab,ti OR 'meta-analysis':ab,ti) AND [article]/lim |
| **Cochrane**: 923 results |
| #1 MeSH descriptor: [Neoplasms] explode all trees  #2 cancer OR carcinoma OR *advanced cancer OR cancer patient  #3 #1 OR #2  #4 (immunotherapy) OR (immune checkpoint inhibitor) OR (nivolumab) OR (pembrolizumab) OR (atezolizumab) OR (PD-1 inhibitor) OR (PD-L1 inhibitor) OR (programmed cell death-1) OR (programmed cell death-ligand 1) OR (cemiplimab) OR (sintilimab) OR (tislelizumab) OR (camrelizumab) OR (durvalumab) OR (avelumab) OR (toripalimab) OR (Ipilimumab) OR (sugemalimab) OR (tremelimumab) OR (Serplulimab) OR (Adebrelimab) OR (Dostarlimab) OR (Cosibelimab) OR (retifanlimab) OR (CTLA-4 inhibitor) OR (cytotoxic T lymphocyte associated antigen-4)  #5 randomized OR randomised  #6 phase NEXT 3  #7 phase NEXT III  #8 #6 OR #7  #9 *first-line OR untreated  #10 drug safety OR adverse drug reaction  #11 real-world OR cost  #12 #3 and #4 and #5 and #8 and #9 and #10 not #11 |
| **Additional search (NEJM Evidence)**: 18 results |
| #1 fulltext:"phase 3" AND (fulltext:immunotherapy OR fulltext:immune OR fulltext:checkpoint OR fulltext:inhibitor OR fulltext:PD-L1 OR fulltext:PD-1 OR fulltext:CTLA-4) AND (articleType:"Original Article")  #2 fulltext:"phase III" AND (fulltext:immunotherapy OR fulltext:immune OR fulltext:checkpoint OR fulltext:inhibitor OR fulltext:PD-L1 OR fulltext:PD-1 OR fulltext:CTLA-4) |

## Supplementary Table 3. Baseline Characteristics of studies included in the additional analysis of the risk of treatment discontinuation due to any AEs or TEAEs

| **Study**  **(First author, year)** | **Indication** | **Treatment group** | **IO agents** | **Number of patients in safety analysis** | **Reported outcomes^a^** | **Median follow-up duration (months)** |
| --- | --- | --- | --- | --- | --- | --- |
| ASTRUM-007 [[1]](https://www.zotero.org/google-docs/?Es9mZJ)  (Song Y, 2023) | ESCC | Chemo-IO | Serplulimab | 382 | TRAE, TEAE | 14.9 |
|  |  | Chemo-only | NA | 168 |  | 14.9 |
| B-FAST [[2]](https://www.zotero.org/google-docs/?2v3oUc)  (Peters S, 2022) | NSCLC | mono-IO | Atezolizumab | 234 | any AE | 18.2^b^ |
|  |  | Chemo-only | NA | 221 |  | 18.2^b^ |
| CAPTAIN-1st [[3]](https://www.zotero.org/google-docs/?g32apl)  (Yang Y, 2021) | NPC | Chemo-IO | Camrelizumab | 134 | TRAE, any AE | 15.6 |
|  |  | Chemo-only | NA | 129 |  | 15.6 |
| CASPIAN [[4]](https://www.zotero.org/google-docs/?nZZ3AM)  (Paz-Ares L, 2019) | SCLC | Chemo-IO | Durvalumab | 265 | any AE | 14.2 |
|  |  | Chemo-only | NA | 266 |  | 14.2 |
| CheckMate 066 [[5]](https://www.zotero.org/google-docs/?dSPmtW) (Robert C, 2015) | NSCLC | mono-IO | Nivolumab | 206 | any AE | 8.9 |
|  |  | Chemo-only | NA | 205 |  | 6.8 |
| CHOICE-01 [[6]](https://www.zotero.org/google-docs/?UOSdg3)  (Wang Z, 2023) | NSCLC | Chemo-IO | Toripalimab | 308 | TEAE | 16.2 |
|  |  | Chemo-only | NA | 156 |  | 16.2 |
| DANUBE [[7]](https://www.zotero.org/google-docs/?as2jsV) (Powles T, 2020) | MIBC | mono-IO | Durvalumab | 345 | TRAE, any AE | 41.2 |
|  |  | dual-IO | Durvalumab,  Tremelimumab | 340 |  | 41.2 |
|  |  | Chemo-only | NA | 313 |  | 41.2 |
| EMPOWER-Lung1 [[8]](https://www.zotero.org/google-docs/?9ggEcc) (Sezer A, 2021) | NSCLC | mono-IO | Cemiplimab | 355 | TEAE | 13.1 |
|  |  | Chemo-only | NA | 342 |  | 13.1 |
| IMpassion130 [[9]](https://www.zotero.org/google-docs/?zgkIQf) (Schmid P, 2018) | Breast | Chemo-IO | Atezolizumab | 452 | any AE | 13 |
|  |  | Chemo-only | NA | 438 |  | 12.5 |
| IMpower110 [[10]](https://www.zotero.org/google-docs/?RVaQRd) (Herbst RS, 2020) | NSCLC | mono-IO | Atezolizumab | 286 | any AE | 13.4 |
|  |  | Chemo-only | NA | 263 |  | 13.4 |
| IMpower130 [[11]](https://www.zotero.org/google-docs/?bEWe6o) (West H, 2019) | NSCLC | Chemo-IO | Atezolizumab | 473 | any AE | 18.5 |
|  |  | Chemo-only | NA | 232 |  | 19.2 |
| IMpower131 [[12]](https://www.zotero.org/google-docs/?HLp01P) (Jotte R, 2020) | NSCLC | Chemo-IO | Atezolizumab | 334 | any AE | 26.8 |
|  |  | Chemo-only | NA | 334 |  | 24.8 |
| IMpower132 [[13]](https://www.zotero.org/google-docs/?G24AeP) (Nishio M, 2021) | NSCLC | Chemo-IO | Atezolizumab | 291 | any AE | 28.4 |
|  |  | Chemo-only | NA | 274 |  | 28.4 |
| IMpower133 [[14]](https://www.zotero.org/google-docs/?FkHQm7) (Horn L, 2018) | SCLC | Chemo-IO | Atezolizumab | 198 | any AE | 13.9 |
|  |  | Chemo-only | NA | 196 |  | 13.9 |
| Imvigor-130 [[15]](https://www.zotero.org/google-docs/?7fmU5Z) (Galsky MD, 2020) | MIBC | mono-IO | Atezolizumab | 354 | any AE | 11.8 |
|  |  | Chemo-IO | Atezolizumab | 453 |  | 11.8 |
|  |  | Chemo-only | NA | 390 |  | 11.8 |
| IPSOS [[16]](https://www.zotero.org/google-docs/?Bnqx2C) (Lee SM, 2023) | NSCLC | mono-IO | Atezolizumab | 300 | any AE | 41 |
|  |  | Chemo-only | NA | 147 |  | 41 |
| JUPITER 06 [[17]](https://www.zotero.org/google-docs/?e8MObG) (Wang ZX, 2022) | ESCC | Chemo-IO | Toripalimab | 257 | TEAE | 7.1 |
|  |  | Chemo-only | NA | 257 |  | 7.1 |
| KEYNOTE-048 [[18]](https://www.zotero.org/google-docs/?9igSeN) (Burtness B, 2019) | HNSCC | mono-IO | Pembrolizumab | 300 | any AE | 11.5 |
|  |  | Chemo-IO | Pembrolizumab | 276 |  | 13.0 |
| KEYNOTE-189 [[19]](https://www.zotero.org/google-docs/?U9FWl5) (Gandhi L, 2018) | NSCLC | Chemo-IO | Pembrolizumab | 405 | any AE | 10.5 |
|  |  | Chemo-only | NA | 202 |  | 10.5 |
| KEYNOTE-361 [[20]](https://www.zotero.org/google-docs/?A0zyep) (Powles T, 2021) | MIBC | mono-IO | Pembrolizumab | 302 | any AE | 31.7 |
|  |  | Chemo-IO | Pembrolizumab | 349 |  | 31.7 |
|  |  | Chemo-only | NA | 342 |  | 31.7 |
| KEYNOTE-407 [[21]](https://www.zotero.org/google-docs/?6ZvPTf) (Paz-Ares L, 2018) | NSCLC | Chemo-IO | Pembrolizumab | 278 | any AE | 7.8 |
|  |  | Chemo-only | NA | 280 |  | 7.8 |
| KEYNOTE-590 [[22]](https://www.zotero.org/google-docs/?JREEes) (Sun JM, 2021) | ESCC | Chemo-IO | Pembrolizumab | 370 | any AE | 22.6 |
|  |  | Chemo-only | NA | 370 |  | 22.6 |
| KEYNOTE-598 [[23]](https://www.zotero.org/google-docs/?hU2Apw) (Boyer M, 2021) | NSCLC | dual-IO | Pembrolizumab, Ipilimumab | 282 | any AE | 20.6 |
|  |  | mono-IO | Pembrolizumab | 281 |  | 20.6 |
| KEYNOTE-604 [[24]](https://www.zotero.org/google-docs/?lZ24SC) (Rudin CM, 2020) | SCLC | Chemo-IO | Pembrolizumab | 223 | any AE | 21.6 |
|  |  | Chemo-only | NA | 223 |  | 21.6 |
| NCT03581786 [[25]](https://www.zotero.org/google-docs/?glIsom) (Mai HQ, 2021) | NPC | Chemo-IO | Toripalimab | 146 | TEAE | 17.9 |
|  |  | Chemo-only | NA | 143 |  | 17.4 |
| ORIENT-11 [[26]](https://www.zotero.org/google-docs/?07XyGt) (Yang Y, 2020) | NSCLC | Chemo-IO | Sintilimab | 266 | any AE | 8.9 |
|  |  | Chemo-only | NA | 131 |  | 8.9 |
| ORIENT-12 [[27]](https://www.zotero.org/google-docs/?qgPhVq) (Zhou C, 2021) | NSCLC | Chemo-IO | Sintilimab | 179 | TEAE | 12.9 |
|  |  | Chemo-only | NA | 178 |  | 12.9 |
| ORIENT-15 [[28]](https://www.zotero.org/google-docs/?fbqgeA) (Lu Z, 2022) | ESCC | Chemo-IO | Sintilimab | 327 | TRAE, TEAE | 16.0 |
|  |  | Chemo-only | NA | 332 |  | 16.9 |
| RATIONALE 304 [[29]](https://www.zotero.org/google-docs/?9hdPjr) (Lu S, 2021) | NSCLC | Chemo-IO | Tislelizumab | 222 | TEAE | 9.8 |
|  |  | Chemo-only | NA | 110 |  | 9.8 |
| RATIONALE 307 [[30]](https://www.zotero.org/google-docs/?wvwuhe) (Wang J, 2021) | NSCLC | Chemo-IO | Tislelizumab | 120 | TEAE | 8.6 |
|  |  | Chemo-only | NA | 117 |  | 8.6 |
| RATIONALE-309 [[31]](https://www.zotero.org/google-docs/?N09yEm) (Yang Y, 2023) | NPC | Chemo-IO | Tislelizumab | 131 | TEAE | 15.5 |
|  |  | Chemo-only | NA | 132 |  | 15.5 |
| RUBY [[32]](https://www.zotero.org/google-docs/?YkkgaY) (Mirza MR, 2023) | Endometrial cancer | Chemo-IO | Dostarlimab | 241 | any AE | 25.4 |
|  |  | Chemo-only | NA | 246 |  | 25.4 |
| TOPAZ-1 [[33]](https://www.zotero.org/google-docs/?hAPgha) (Oh DY, 2022) | BTC | Chemo-IO | Durvalumab | 338 | TRAE, any AE | 16.8 |
|  |  | Chemo-only | NA | 342 |  | 15.9 |

^a^Types of AEs leading to treatment discontinuation

^b^Only reported follow-up duration in patients with bTMB ≥16 (n=145)

AE indicates adverse event; BTC, biliary tract cancer; bTMB, blood tumor mutation burden; Chemo-dual-IO, dual-immunotherapy combined with chemotherapy; Chemo-IO, mono-immunotherapy combined with chemotherapy; Chemo-only, chemotherapy alone; dual-IO, dual-immunotherapy; ESCC, esophageal squamous cell carcinoma; GI, gastrointestinal; HCC, hepatocellular carcinoma; HNSCC, head and neck squamous cell carcinoma; IO, immunotherapy; MIBC, muscle invasive bladder cancer; mono-IO, mono-immunotherapy; NA, not applicable; NPC, Nasopharyngeal carcinoma; NSCLC, non-small cell lung cancer; SCLC, small cell lung cancer; TEAE, treatment emergent adverse event; TRAE, treatment-related adverse event;

## Supplementary Figure 1. Assessment of Risk of Bias

(A) For studies that reported discontinuation due to TRAEs. (B) For studies that reported discontinuation due to any AE/TEAEs.

The risk of bias was assessed using the Cochrane Collaboration's tool (2.0). AE indicates adverse event; TEAE, treatment emergent adverse event; TRAE, treatment-related adverse event.


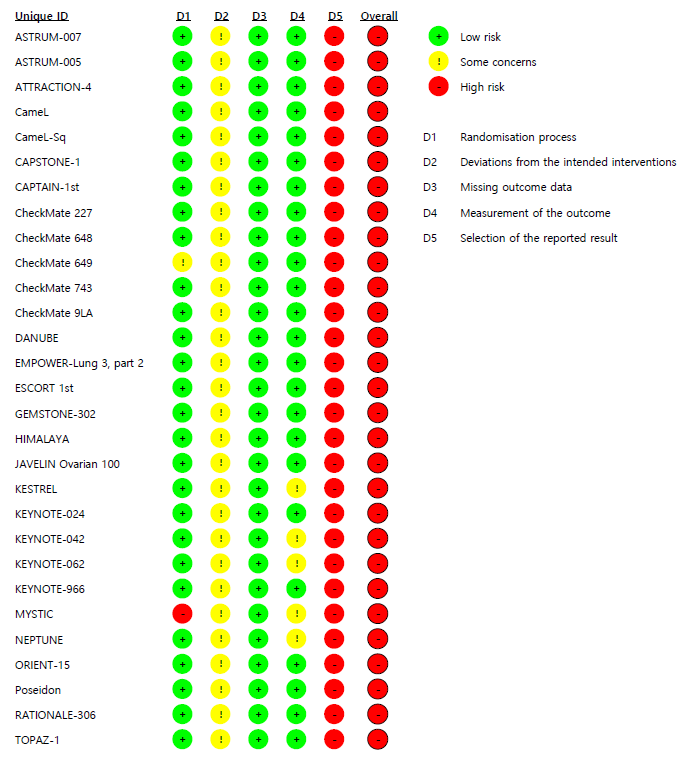
**(A)**

##
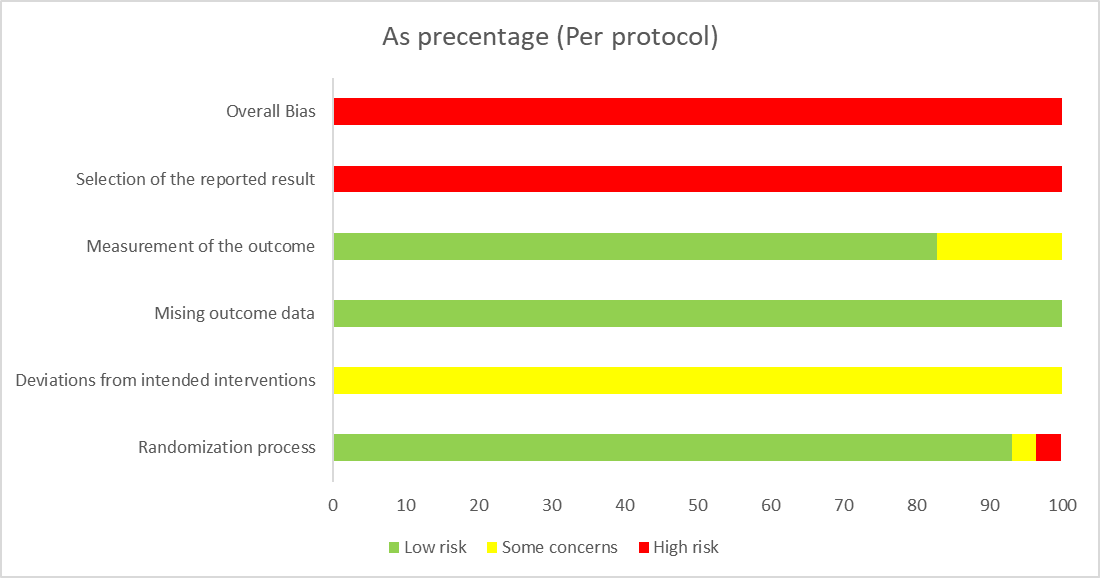


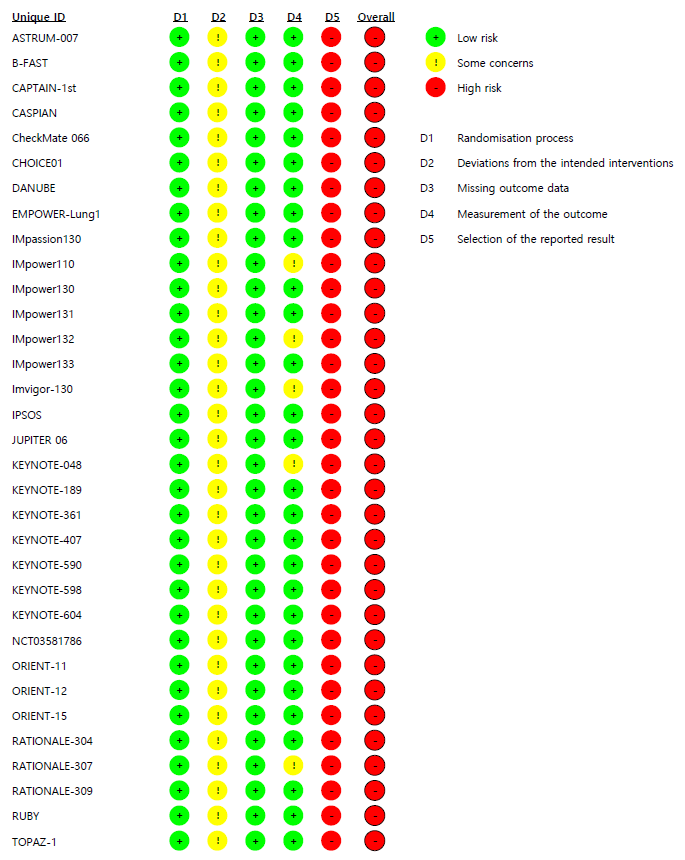
**(B)**

##
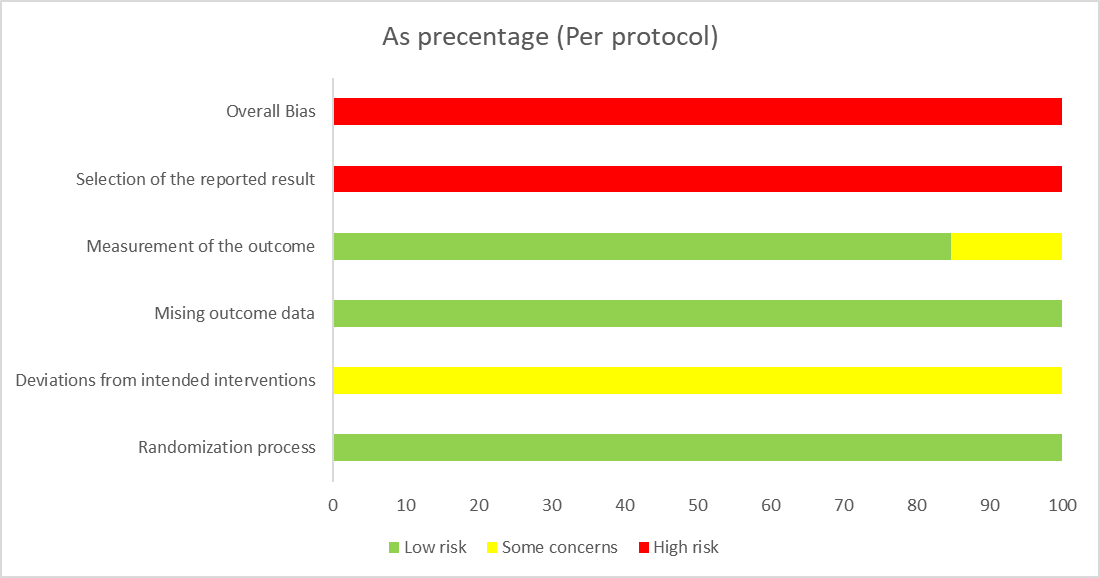


## Supplementary Figure 2. Assessment of Publication Bias

(A) Funnel plot for studies reporting discontinuation due to TRAEs in various cancer types. (B) Funnel plot for studies reporting discontinuation due to TRAEs in NSCLC.

(C) Funnel plot for studies reporting discontinuation due to any AE/TEAEs in various cancer types. (D) Funnel plot for studies reporting discontinuation due to any AE/TEAEs in NSCLC.

Each figure's P-value was determined using Egger’s test. AE indicates adverse event; Chemo-dual-IO, dual-immunotherapy combined with chemotherapy; Chemo-IO, mono-immunotherapy combined with chemotherapy; Chemo-only, chemotherapy alone; dual-IO, dual-immunotherapy; mono-IO, mono-immunotherapy; NSCLC, non-small cell lung cancer; TEAE, treatment emergent adverse event; TRAE, treatment-related adverse event.

**(A) (B)**
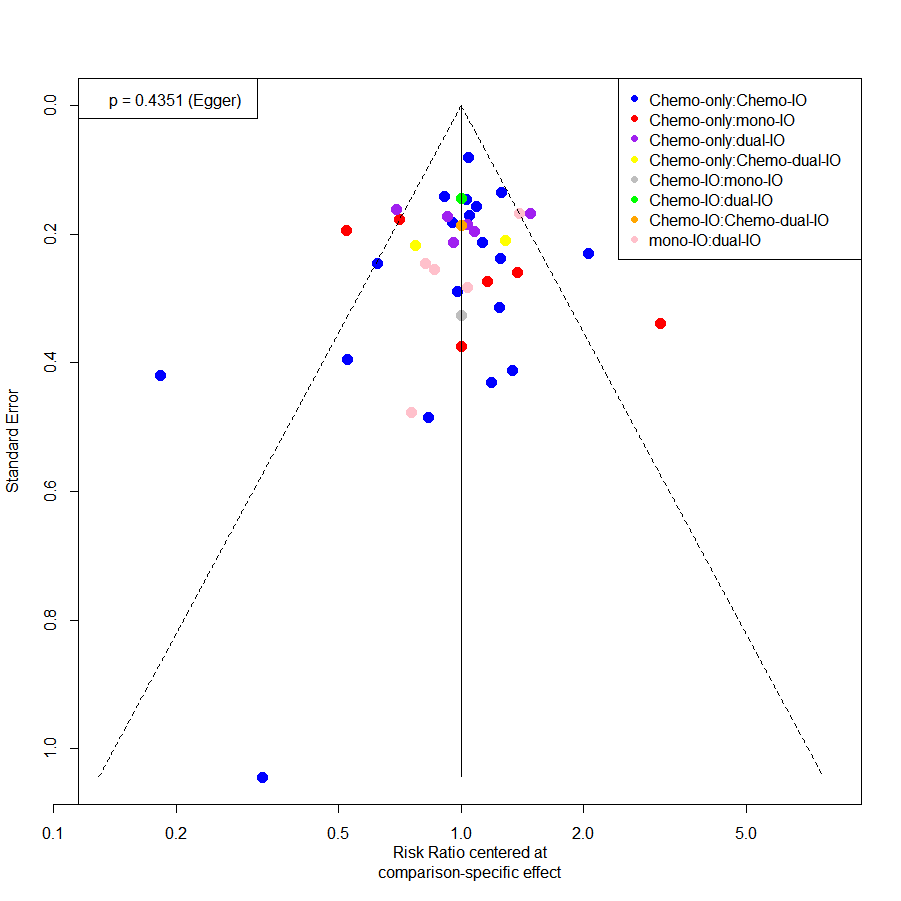


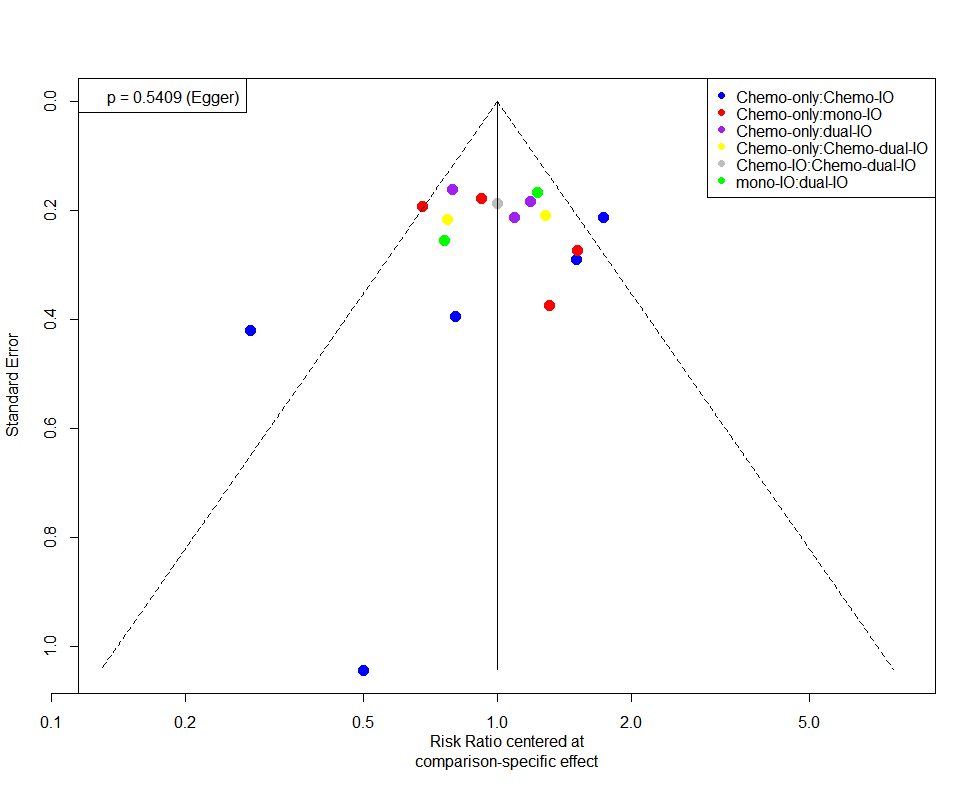


##

**(C) (D)**
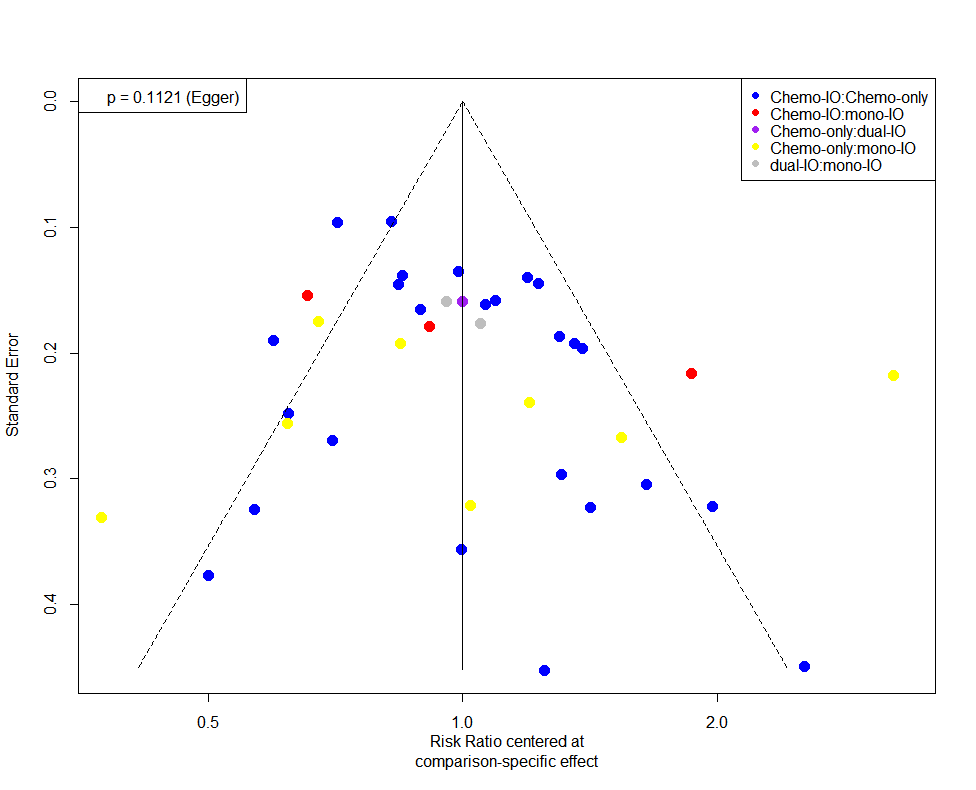

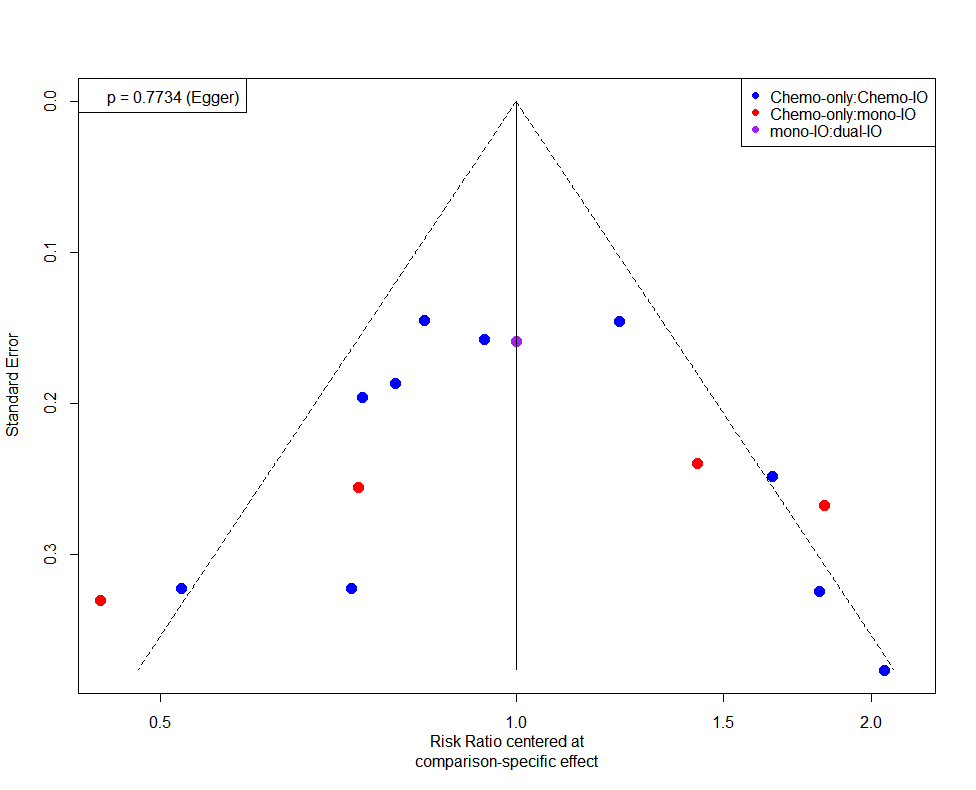


## Supplementary Figure 3. Network plot illustrating comparisons of relative risks (RRs) of discontinuation due to any AEs or TEAEs among different treatment regimens

(A) For various cancer types, (B) For NSCLC subgroup

The width of lines is proportional to the number of trials that compare directly in the same trial.

Chemo-IO indicates mono-immunotherapy combined with chemotherapy; Chemo-only, chemotherapy alone; dual-IO, dual-immunotherapy; mono-IO, mono-immunotherapy; NSCLC, non-small cell lung cancer.

**(A) (B)**
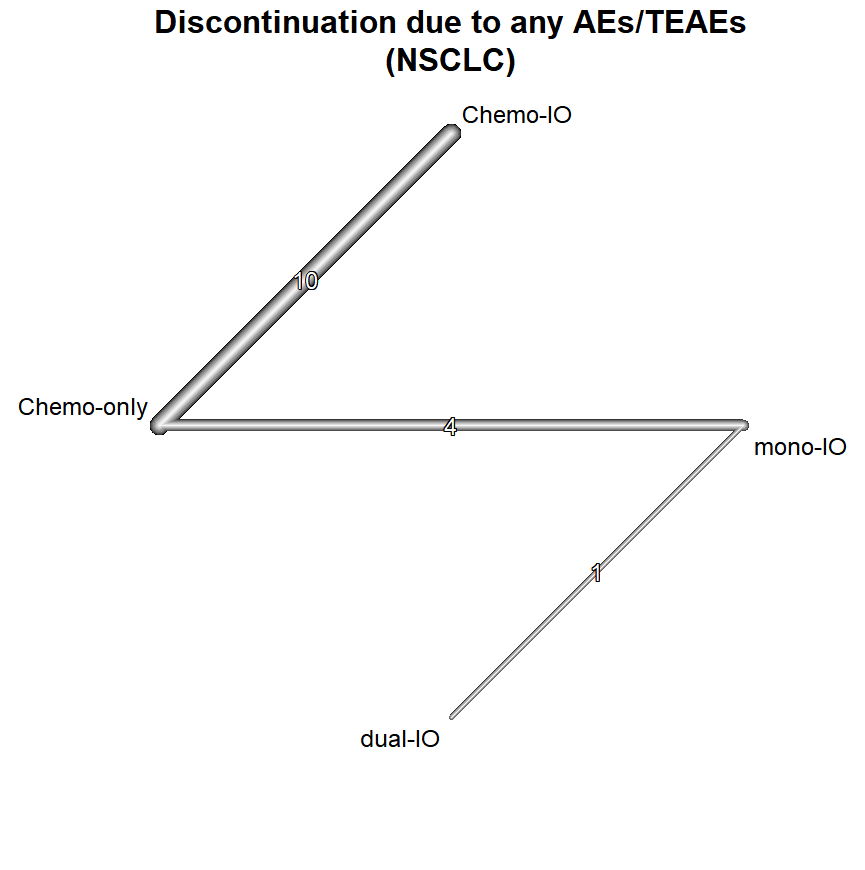

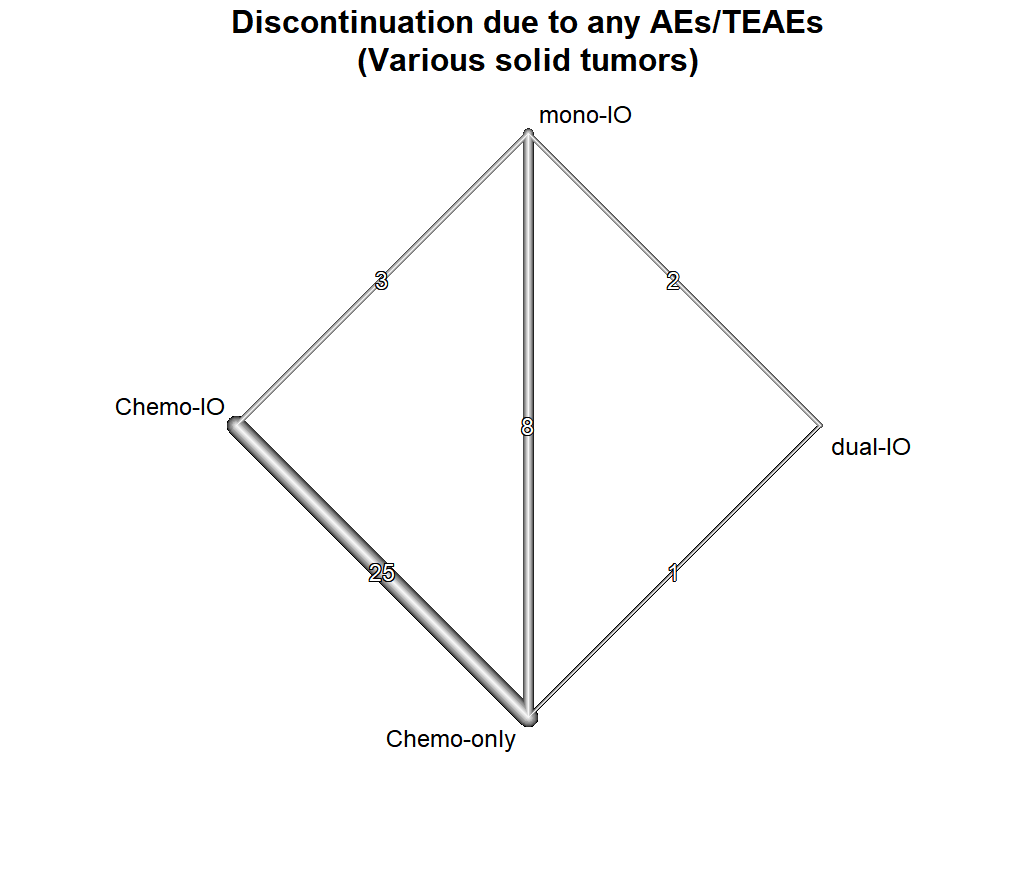


##

## Supplementary Figure 4. Relative risks (RRs) of discontinuation due to any AEs or TEAEs across various solid tumors

(A) Forest plot of RR of treatment discontinuation due to any AEs or TEAEs. (B) Direct and overall comparisons of discontinuation due to any AEs or TEAEs. Each cell contains pooled RR with 95% CI. And RR more than 1 means upper-row treatment has more risk of treatment discontinuation. The overall comparison outcomes, which combine direct and indirect comparison results from the network meta-analysis are displayed in the left lower half, while results from pairwise meta-analysis are shown in the right upper half, if available. Significant results are in bold. Chemo-dual-IO indicates dual-immunotherapy combined with chemotherapy; Chemo-IO, mono-immunotherapy combined with chemotherapy; Chemo-only, chemotherapy alone; CI, confidence interval; dual-IO, dual-immunotherapy; mono-IO, mono-immunotherapy; NA, not available; NSCLC, non-small cell lung cancer; RR, relative risk; TRAE, treatment-related adverse event.

**(A) (B)**
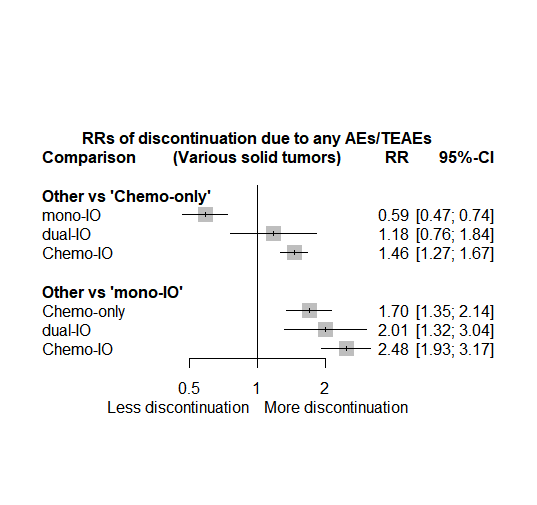

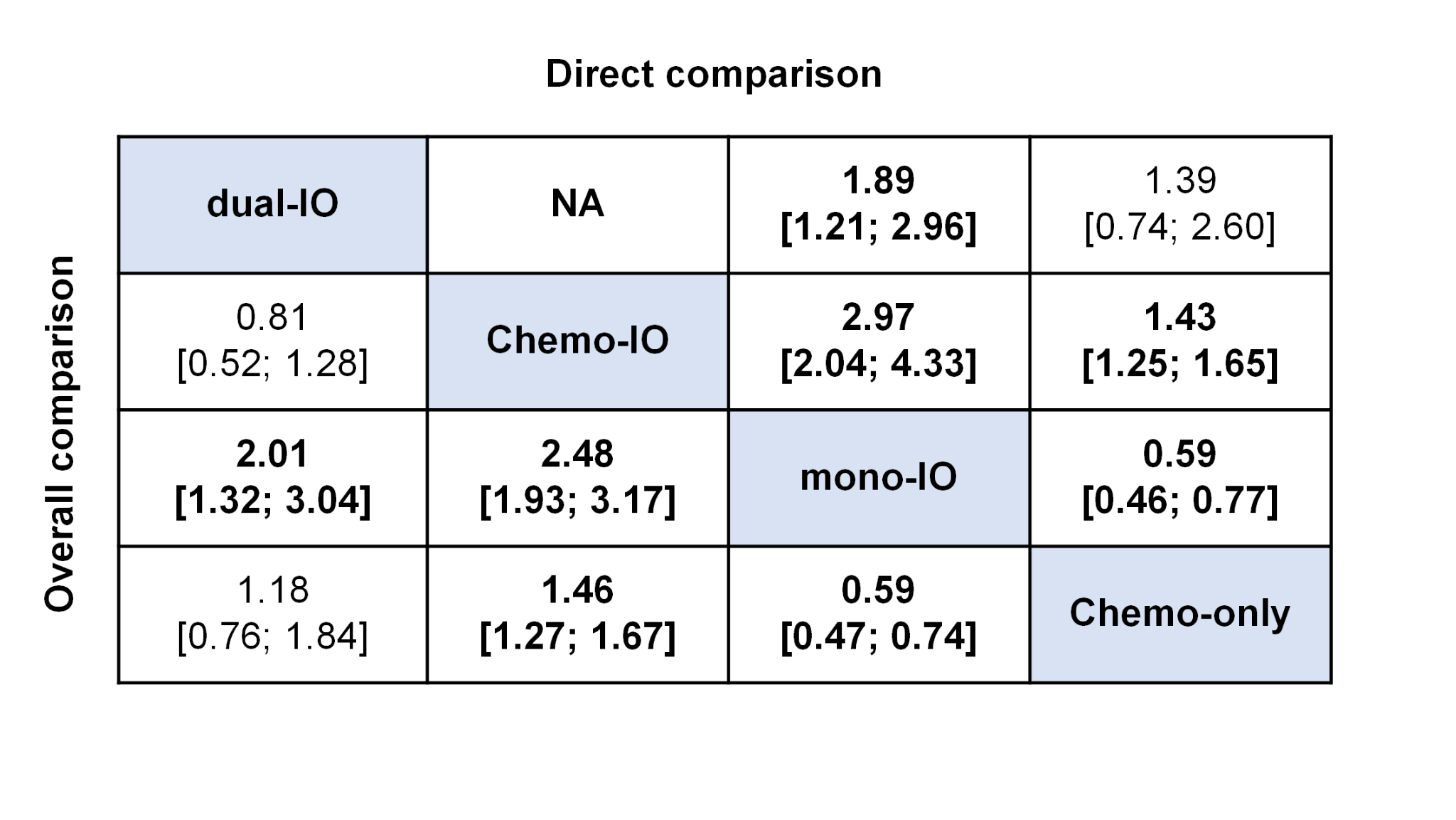


## Supplementary Figure 5. Relative risks (RRs) of discontinuation due to any AEs or TEAEs in NSCLC

(A) Forest plot of RR of treatment discontinuation due to TRAEs. (B) Direct and overall comparisons of discontinuation due to TRAEs. Each cell contains pooled RR with 95% CI. And RR more than 1 means upper-row treatment has more risk of treatment discontinuation. The overall comparison outcomes, which combine direct and indirect comparison results from the network meta-analysis are displayed in the left lower half, while results from pairwise meta-analysis are shown in the right upper half, if available. Significant results are in bold. Chemo-dual-IO indicates dual-immunotherapy combined with chemotherapy; Chemo-IO, mono-immunotherapy combined with chemotherapy; Chemo-only, chemotherapy alone; CI, confidence interval; dual-IO, dual-immunotherapy; mono-IO, mono-immunotherapy; NA, not available; NSCLC, non-small cell lung cancer; RR, relative risk; TRAE, treatment-related adverse event.

**(A) (B)**
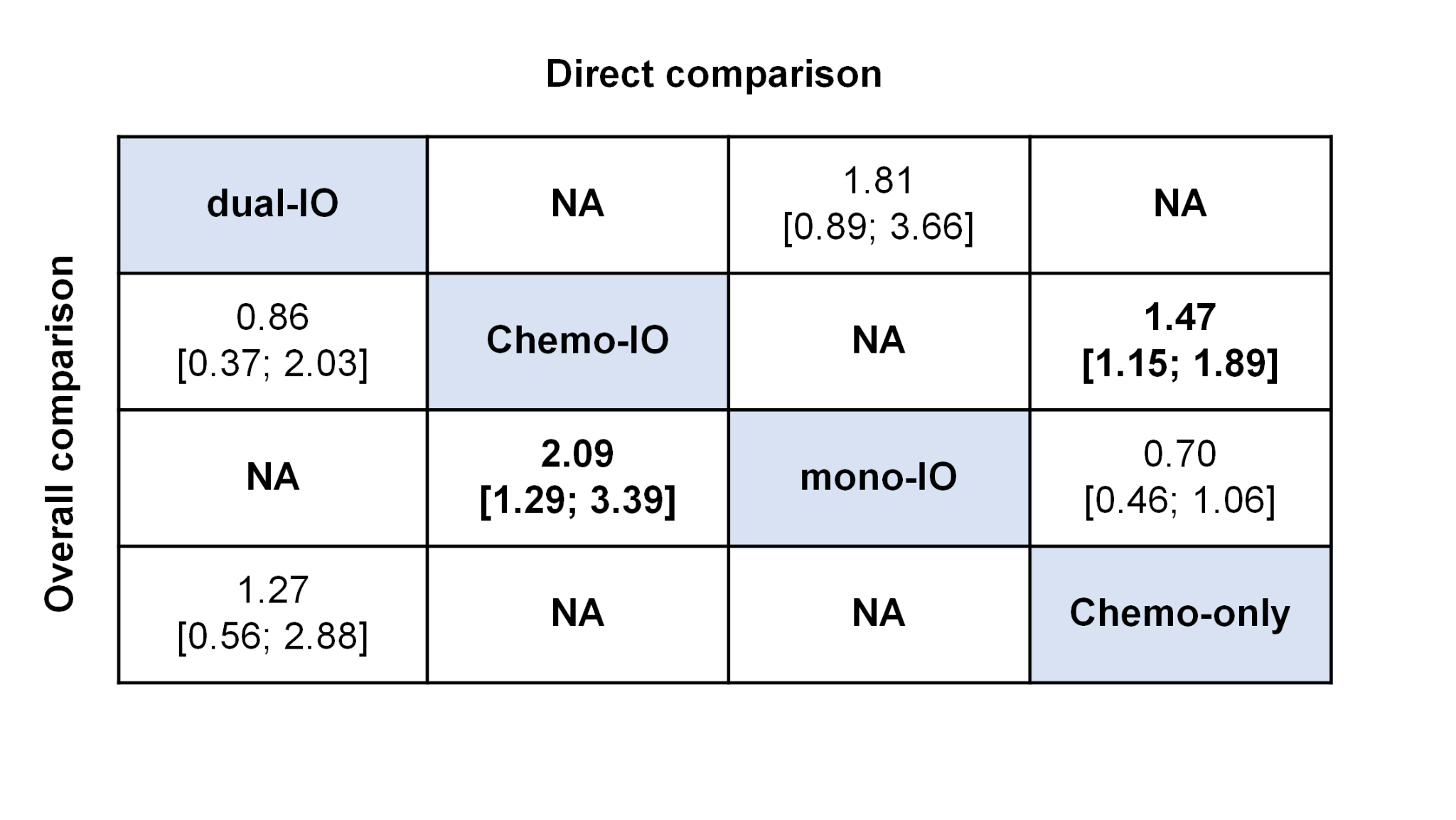


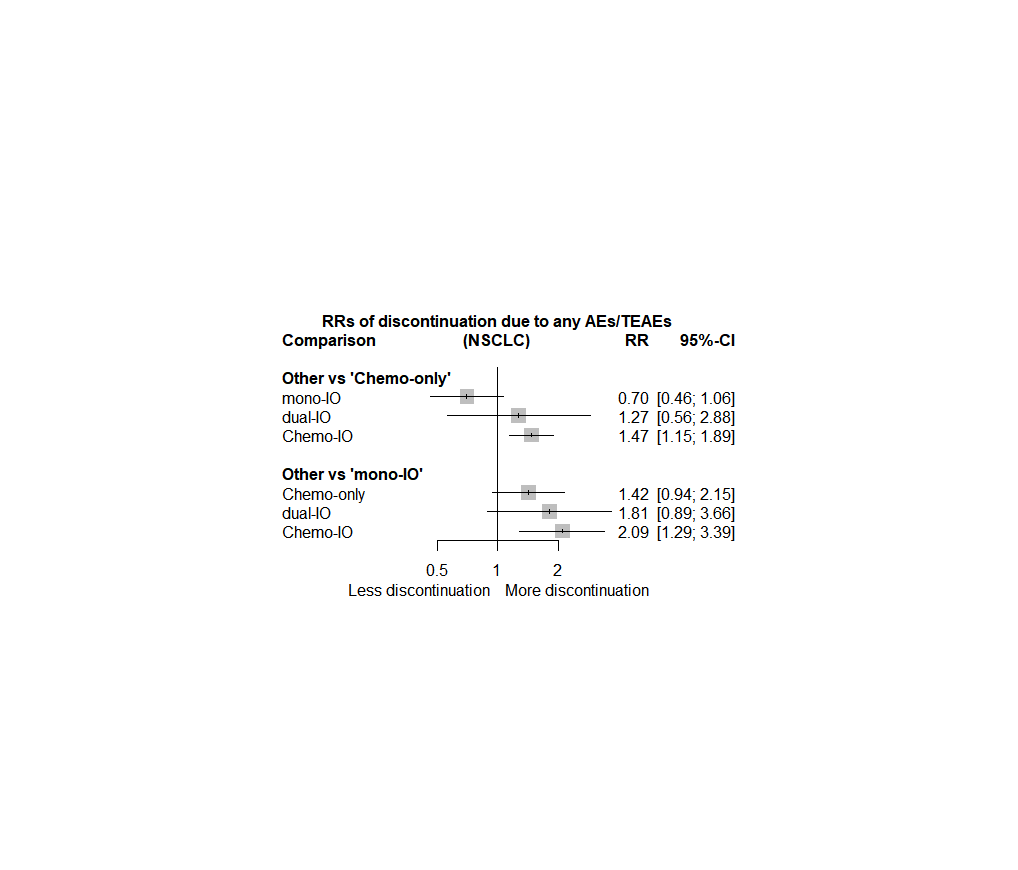


**Supplementary References**

[1. Song Y, Zhang B, Xin D, Kou X, Tan Z, Zhang S, et al. First-line serplulimab or placebo plus chemotherapy in PD-L1-positive esophageal squamous cell carcinoma: a randomized, double-blind phase 3 trial. Nat Med. 2023;29:473–82.](https://www.zotero.org/google-docs/?Bzj5SQ)

[2. Peters S, Dziadziuszko R, Morabito A, Felip E, Gadgeel SM, Cheema P, et al. Atezolizumab versus chemotherapy in advanced or metastatic NSCLC with high blood-based tumor mutational burden: primary analysis of BFAST cohort C randomized phase 3 trial. Nat Med. 2022;28:1831–9.](https://www.zotero.org/google-docs/?Bzj5SQ)

[3. Yang Y, Qu S, Li J, Hu C, Xu M, Li W, et al. Camrelizumab versus placebo in combination with gemcitabine and cisplatin as first-line treatment for recurrent or metastatic nasopharyngeal carcinoma (CAPTAIN-1st): a multicentre, randomised, double-blind, phase 3 trial. Lancet Oncol. 2021;22:1162–74.](https://www.zotero.org/google-docs/?Bzj5SQ)

[4. Paz-Ares L, Dvorkin M, Chen Y, Reinmuth N, Hotta K, Trukhin D, et al. Durvalumab plus platinum–etoposide versus platinum–etoposide in first-line treatment of extensive-stage small-cell lung cancer (CASPIAN): a randomised, controlled, open-label, phase 3 trial. Lancet. 2019;394:1929–39.](https://www.zotero.org/google-docs/?Bzj5SQ)

[5. Robert C, Long GV, Brady B, Dutriaux C, Maio M, Mortier L, et al. Nivolumab in previously untreated melanoma without BRAF mutation. N Engl J Med. 2015;372:320–30.](https://www.zotero.org/google-docs/?Bzj5SQ)

[6. Wang Z, Wu L, Li B, Cheng Y, Li X, Wang X, et al. Toripalimab Plus Chemotherapy for Patients With Treatment-Naive Advanced Non-Small-Cell Lung Cancer: A Multicenter Randomized Phase III Trial (CHOICE-01). J Clin Oncol. 2023;41:651–63.](https://www.zotero.org/google-docs/?Bzj5SQ)

[7. Powles T, van der Heijden MS, Castellano D, Galsky MD, Loriot Y, Petrylak DP, et al. Durvalumab alone and durvalumab plus tremelimumab versus chemotherapy in previously untreated patients with unresectable, locally advanced or metastatic urothelial carcinoma (DANUBE): a randomised, open-label, multicentre, phase 3 trial. Lancet Oncol. 2020;21:1574–88.](https://www.zotero.org/google-docs/?Bzj5SQ)

[8. Sezer A, Kilickap S, Gümüş M, Bondarenko I, Özgüroğlu M, Gogishvili M, et al. Cemiplimab monotherapy for first-line treatment of advanced non-small-cell lung cancer with PD-L1 of at least 50%: a multicentre, open-label, global, phase 3, randomised, controlled trial. Lancet. 2021;397:592–604.](https://www.zotero.org/google-docs/?Bzj5SQ)

[9. Schmid P, Adams S, Rugo HS, Schneeweiss A, Barrios CH, Iwata H, et al. Atezolizumab and Nab-Paclitaxel in Advanced Triple-Negative Breast Cancer. N Engl J Med. 2018;379:2108–21.](https://www.zotero.org/google-docs/?Bzj5SQ)

[10. Herbst RS, Giaccone G, de Marinis F, Reinmuth N, Vergnenegre A, Barrios CH, et al. Atezolizumab for First-Line Treatment of PD-L1-Selected Patients with NSCLC. N Engl J Med. 2020;383:1328–39.](https://www.zotero.org/google-docs/?Bzj5SQ)

[11. West H, McCleod M, Hussein M, Morabito A, Rittmeyer A, Conter HJ, et al. Atezolizumab in combination with carboplatin plus nab-paclitaxel chemotherapy compared with chemotherapy alone as first-line treatment for metastatic non-squamous non-small-cell lung cancer (IMpower130): a multicentre, randomised, open-label, phase 3 trial. Lancet Oncol. 2019;20:924–37.](https://www.zotero.org/google-docs/?Bzj5SQ)

[12. Jotte R, Cappuzzo F, Vynnychenko I, Stroyakovskiy D, Rodríguez-Abreu D, Hussein M, et al. Atezolizumab in Combination With Carboplatin and Nab-Paclitaxel in Advanced Squamous NSCLC (IMpower131): Results From a Randomized Phase III Trial. J Thorac Oncol. 2020;15:1351–60.](https://www.zotero.org/google-docs/?Bzj5SQ)

[13. Nishio M, Barlesi F, West H, Ball S, Bordoni R, Cobo M, et al. Atezolizumab Plus Chemotherapy for First-Line Treatment of Nonsquamous NSCLC: Results From the Randomized Phase 3 IMpower132 Trial. J Thorac Oncol. 2021;16:653–64.](https://www.zotero.org/google-docs/?Bzj5SQ)

[14. Horn L, Mansfield AS, Szczęsna A, Havel L, Krzakowski M, Hochmair MJ, et al. First-Line Atezolizumab plus Chemotherapy in Extensive-Stage Small-Cell Lung Cancer. N Engl J Med. 2018;379:2220–9.](https://www.zotero.org/google-docs/?Bzj5SQ)

[15. Galsky MD, Arija JÁA, Bamias A, Davis ID, De Santis M, Kikuchi E, et al. Atezolizumab with or without chemotherapy in metastatic urothelial cancer (IMvigor130): a multicentre, randomised, placebo-controlled phase 3 trial. Lancet. 2020;395:1547–57.](https://www.zotero.org/google-docs/?Bzj5SQ)

[16. Lee SM, Schulz C, Prabhash K, Kowalski D, Szczesna A, Han B, et al. First-line atezolizumab monotherapy versus single-agent chemotherapy in patients with non-small-cell lung cancer ineligible for treatment with a platinum-containing regimen (IPSOS): a phase 3, global, multicentre, open-label, randomised controlled study. Lancet. 2023;:S0140-6736(23)00774-2.](https://www.zotero.org/google-docs/?Bzj5SQ)

[17. Wang Z-X, Cui C, Yao J, Zhang Y, Li M, Feng J, et al. Toripalimab plus chemotherapy in treatment-naïve, advanced esophageal squamous cell carcinoma (JUPITER-06): A multi-center phase 3 trial. Cancer Cell. 2022;40:277-288.e3.](https://www.zotero.org/google-docs/?Bzj5SQ)

[18. Burtness B, Harrington KJ, Greil R, Soulières D, Tahara M, de Castro G, et al. Pembrolizumab alone or with chemotherapy versus cetuximab with chemotherapy for recurrent or metastatic squamous cell carcinoma of the head and neck (KEYNOTE-048): a randomised, open-label, phase 3 study. Lancet. 2019;394:1915–28.](https://www.zotero.org/google-docs/?Bzj5SQ)

[19. Gandhi L, Rodríguez-Abreu D, Gadgeel S, Esteban E, Felip E, De Angelis F, et al. Pembrolizumab plus Chemotherapy in Metastatic Non–Small-Cell Lung Cancer. N Engl J Med. 2018;378:2078–92.](https://www.zotero.org/google-docs/?Bzj5SQ)

[20. Powles T, Csőszi T, Özgüroğlu M, Matsubara N, Géczi L, Cheng SY-S, et al. Pembrolizumab alone or combined with chemotherapy versus chemotherapy as first-line therapy for advanced urothelial carcinoma (KEYNOTE-361): a randomised, open-label, phase 3 trial. Lancet Oncol. 2021;22:931–45.](https://www.zotero.org/google-docs/?Bzj5SQ)

[21. Paz-Ares L, Luft A, Vicente D, Tafreshi A, Gümüş M, Mazières J, et al. Pembrolizumab plus Chemotherapy for Squamous Non–Small-Cell Lung Cancer. N Engl J Med. 2018;379:2040–51.](https://www.zotero.org/google-docs/?Bzj5SQ)

[22. Sun J-M, Shen L, Shah MA, Enzinger P, Adenis A, Doi T, et al. Pembrolizumab plus chemotherapy versus chemotherapy alone for first-line treatment of advanced oesophageal cancer (KEYNOTE-590): a randomised, placebo-controlled, phase 3 study. Lancet. 2021;398:759–71.](https://www.zotero.org/google-docs/?Bzj5SQ)

[23. Boyer M, Şendur MAN, Rodríguez-Abreu D, Park K, Lee DH, Çiçin I, et al. Pembrolizumab Plus Ipilimumab or Placebo for Metastatic Non-Small-Cell Lung Cancer With PD-L1 Tumor Proportion Score ≥ 50%: Randomized, Double-Blind Phase III KEYNOTE-598 Study. J Clin Oncol. 2021;39:2327–38.](https://www.zotero.org/google-docs/?Bzj5SQ)

[24. Rudin CM, Awad MM, Navarro A, Gottfried M, Peters S, Csőszi T, et al. Pembrolizumab or Placebo Plus Etoposide and Platinum as First-Line Therapy for Extensive-Stage Small-Cell Lung Cancer: Randomized, Double-Blind, Phase III KEYNOTE-604 Study. J Clin Oncol. 2020;38:2369–79.](https://www.zotero.org/google-docs/?Bzj5SQ)

[25. Mai H-Q, Chen Q-Y, Chen D, Hu C, Yang K, Wen J, et al. Toripalimab or placebo plus chemotherapy as first-line treatment in advanced nasopharyngeal carcinoma: a multicenter randomized phase 3 trial. Nat Med. 2021;27:1536–43.](https://www.zotero.org/google-docs/?Bzj5SQ)

[26. Yang Y, Wang Z, Fang J, Yu Q, Han B, Cang S, et al. Efficacy and Safety of Sintilimab Plus Pemetrexed and Platinum as First-Line Treatment for Locally Advanced or Metastatic Nonsquamous NSCLC: a Randomized, Double-Blind, Phase 3 Study (Oncology pRogram by InnovENT anti-PD-1-11). J Thorac Oncol. 2020;15:1636–46.](https://www.zotero.org/google-docs/?Bzj5SQ)

[27. Zhou C, Wu L, Fan Y, Wang Z, Liu L, Chen G, et al. Sintilimab Plus Platinum and Gemcitabine as First-Line Treatment for Advanced or Metastatic Squamous NSCLC: Results From a Randomized, Double-Blind, Phase 3 Trial (ORIENT-12). J Thorac Oncol. 2021;16:1501–11.](https://www.zotero.org/google-docs/?Bzj5SQ)

[28. Lu Z, Wang J, Shu Y, Liu L, Kong L, Yang L, et al. Sintilimab versus placebo in combination with chemotherapy as first line treatment for locally advanced or metastatic oesophageal squamous cell carcinoma (ORIENT-15): multicentre, randomised, double blind, phase 3 trial. BMJ. 2022;377:e068714.](https://www.zotero.org/google-docs/?Bzj5SQ)

[29. Lu S, Wang J, Yu Y, Yu X, Hu Y, Ai X, et al. Tislelizumab Plus Chemotherapy as First-Line Treatment for Locally Advanced or Metastatic Nonsquamous NSCLC (RATIONALE 304): A Randomized Phase 3 Trial. J Thorac Oncol. 2021;16:1512–22.](https://www.zotero.org/google-docs/?Bzj5SQ)

[30. Wang J, Lu S, Yu X, Hu Y, Sun Y, Wang Z, et al. Tislelizumab Plus Chemotherapy vs Chemotherapy Alone as First-line Treatment for Advanced Squamous Non–Small-Cell Lung Cancer: A Phase 3 Randomized Clinical Trial. JAMA Oncol. 2021;7:709–17.](https://www.zotero.org/google-docs/?Bzj5SQ)

[31. Yang Y, Pan J, Wang H, Zhao Y, Qu S, Chen N, et al. Tislelizumab plus chemotherapy as first-line treatment for recurrent or metastatic nasopharyngeal cancer: A multicenter phase 3 trial (RATIONALE-309). Cancer Cell. 2023;41:1061-1072.e4.](https://www.zotero.org/google-docs/?Bzj5SQ)

[32. Mirza MR, Chase DM, Slomovitz BM, dePont Christensen R, Novák Z, Black D, et al. Dostarlimab for Primary Advanced or Recurrent Endometrial Cancer. N Engl J Med. 2023;388:2145–58.](https://www.zotero.org/google-docs/?Bzj5SQ)

[33. Oh D-Y, Ruth He A, Qin S, Chen L-T, Okusaka T, Vogel A, et al. Durvalumab plus Gemcitabine and Cisplatin in Advanced Biliary Tract Cancer. NEJM Evid. 2022;1:EVIDoa2200015.](https://www.zotero.org/google-docs/?Bzj5SQ)
